# Sex and the Single Cell. II. There Is a Time and Place for Sex

**DOI:** 10.1371/journal.pbio.1000365

**Published:** 2010-05-04

**Authors:** Carmen C. Robinett, Alexander G. Vaughan, Jon-Michael Knapp, Bruce S. Baker

**Affiliations:** 1 Biology Department, Stanford University, Stanford, California, United States of America; 2 Neuroscience Program, Stanford University, Stanford, California, United States of America; Stowers Institute for Medical Research, United States of America

## Abstract

The *Drosophila melanogaster* sex hierarchy controls sexual differentiation of somatic cells via the activities of the terminal genes in the hierarchy, *doublesex* (*dsx*) and *fruitless* (*fru*). We have targeted an insertion of *GAL4* into the *dsx* gene, allowing us to visualize *dsx*-expressing cells in both sexes. Developmentally and as adults, we find that both *XX* and *XY* individuals are fine mosaics of cells and tissues that express *dsx* and/or *fruitless (fru^M^)*, and hence have the potential to sexually differentiate, and those that don't. Evolutionary considerations suggest such a mosaic expression of sexuality is likely to be a property of other animal species having two sexes. These results have also led to a major revision of our view of how sex-specific functions are regulated by the sex hierarchy in flies. Rather than there being a single regulatory event that governs the activities of all downstream sex determination regulatory genes—turning on *Sex lethal (Sxl)* RNA splicing activity in females while leaving it turned off in males—there are, in addition, elaborate temporal and spatial transcriptional controls on the expression of the terminal regulatory genes, *dsx* and *fru*. Thus tissue-specific aspects of sexual development are jointly specified by post-transcriptional control by *Sxl* and by the transcriptional controls of *dsx* and *fru* expression.

## Abstract 2

In both male and female *Drosophila*, only a subset of cells have the potential to sexually differentiate, making both males and females mosaics of sexually differentiated and sexually undifferentiated cells.

## Author Summary

Morphologically, fruit flies are either male or female. The specification of sex is a multi-step process that depends on whether the fertilized egg has only one X chromosome (will develop as male) or two X chromosomes (will develop as female). This initial assessment of sex activates a cascade of regulatory genes that ultimately results in expression of either the male or female version of the protein encoded by the *doublesex* gene (*dsx*). These sex-specific proteins from the *dsx* gene direct most aspects of somatic sexual development, including the development of all of the secondary sexual characteristics that visibly distinguish males and females. In flies, as in most animal species, only some tissues are obviously different between the two sexes, so we asked the question of whether all cells in the animal nevertheless know which sex they are. That is, do all cells express *dsx*? We have developed a genetic tool that lets us visualize the cells in which the *dsx* is expressed. Strikingly, *dsx* is only expressed in a subset of tissues. Thus, adult flies of both sexes appear to be mosaics of cells that do know their sex and cells that do not know their sex.

## Introduction

A single, multi-branched regulatory hierarchy specifies all somatic sexual differences in *Drosophila melanogaster* [1]–[5]. The fly sex hierarchy is of great intrinsic interest as a model developmental system to dissect both how information is passed through a molecular network and how the actions of the terminal regulatory genes in that network are coordinated with the actions of other patterning hierarchies to orchestrate sex-specific aspects of development, morphogenesis, differentiation, and adult functions.

One outstanding issue with respect to the functioning of the sex hierarchy in flies is whether all cells are sexually differentiated. While the different roles of the two sexes in courtship and reproduction have led to the evolution of the arrays of female- and male-specific features that characterize the two sexes, in flies, as in most animal species, these overt sexual differences are limited to subsets of tissues. Nonetheless, we tend to think of “femaleness” and “maleness” as two distinct states of being that pervade individuals (certainly with respect to humans, and we would submit this viewpoint carries over to color our thinking of sexuality in other species as well). However, from a developmental point of view, there is a question as to whether tissues and organs that are not discernibly different between males and females “know” their sex (i.e., express and utilize the final regulatory genes in the hierarchy) and as a consequence differ sexually in subtle ways between males and females, or alternatively whether males and females are really sexual mosaics in which some cells know their sex and differentiate sex-appropriately, while other cells do not know their sex and thus differentiate identically in males and females.

**Figure 1:**
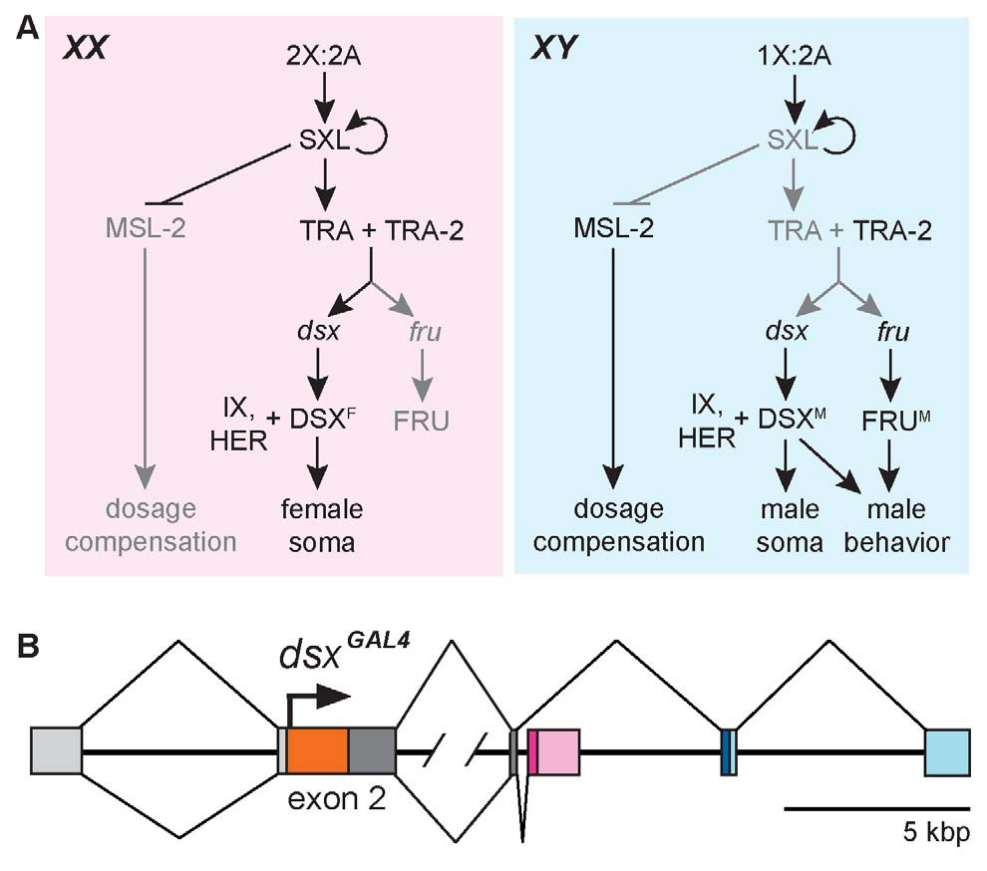
The Drosophila sex hierarchy. (A) The sex determination hierarchy in Drosophila regulates all sexually dimorphic aspects of development. The ratio of X chromosomes to autosomes determines the RNA splicing activity of SXL, which leads to sex-specific alternative splicing of *dsx* and *fru* transcripts at the bottom of the hierarchy. The resulting male- and female-specific DSX and FRU isoforms confer sexual identity to the cells in which they are produced. (Adapted from [2].) (B) The *dsx^GAL4^* gene. Targeted insertion of *GAL4* coding sequence (orange box) after the translational start codon (arrow) of *dsx* in exon 2 allows expression of GAL4 wherever *dsx* is expressed. Chromosomal sequences are color-coded as follows: 5′ UTR sequences common to the mRNAs of both sexes (light gray), N-terminal coding sequence common to mRNAs of both sexes (dark gray), coding and 3′ UTR sequences specific to females (magenta and pink, respectively), coding and 3′ UTR sequences specific to males (dark and light blue, respectively), and intronic sequences (black line). Male-specific and female-specific splicing patterns are depicted above or below the chromosome, respectively. Slash marks in the second intron represent ∼24 kb of DNA.

The three branches of the fly sex hierarchy (Figure 1A) govern: (1) X chromosome dosage compensation (reviews [1],[6]), (2) male sexual and aggressive behaviors via the action of the *fruitless* (*fru*) gene in the nervous system (reviews [4],[5],[7]–[9]), and (3) all other somatic sexual differences via the action of the *doublesex* (*dsx*) gene (which also functions in the nervous system; see below) (reviews [1],[2],[5],[10]). The initial steps in somatic sex determination assess the X chromosome∶autosome ratio and establish sex by setting the RNA splicing activity encoded by *Sex lethal* (*Sxl*) to “ON” in females (*XX*) and “OFF” in males (*XY*). Once turned ON, SXL activity in females is maintained by a positive autoregulatory feedback loop. In females, SXL also blocks translation of *msl-2* mRNA and thus prevents dosage compensation. In addition, SXL directs the female-specific splicing of transcripts from the *transformer (tra)* gene. The female-specific TRA protein together with the TRA-2 protein directs female-specific splicing of pre-mRNAs arising from the *dsx* and the P1 *fruitless (fru^M^)* promoters to generate female-specific *dsx* and *fru* mRNAs. In males, there is no SXL activity, and so (1) dosage compensation occurs and (2) *tra* transcripts containing premature stop codons are produced by the default-splicing pathway. The absence of TRA protein in males leads to the default splicing of *dsx* and P1 promoter-derived *fru* transcripts to produce male-specific mRNAs. The female- and male-specific *dsx* transcripts both encode proteins (DSX^F^ and DSX^M^, respectively), whereas only the P1 promoter-derived *fru* male-specific transcripts are translated and they encode FRU^M^ protein.

The widely accepted view has been that regulatory genes of the Drosophila sex hierarchy are expressed ubiquitously in the soma and that all cells “know” their sex. In particular, it was shown that *Sxl* is expressed in all somatic cells [11], which made sense since *Sxl* is needed to make dosage compensation sex-specific throughout the soma (reviews [12]–). Surprisingly, *tra-2* and *ix* are expressed in both males and females, although they function in sexual development only in females, leading to the proposals that these genes were also ubiquitously expressed [16]–[18]. Finally, as the sex-specific splicing of *dsx* and *tra* pre-mRNAs was sufficient to account for all known roles of these genes, it was inferred that they too were ubiquitously expressed.

However, emerging evidence indicated that the terminal genes in the hierarchy, *fru* and *dsx*, are not expressed ubiquitously. Expression of *fru^M^* is restricted to subsets of neurons in the central and peripheral nervous systems [19]–[22]. Evidence suggesting that expression of *dsx* is also spatially and temporally restricted came first from the finding that expression of *dsx* mRNA is restricted to the gonad in embryos (www.fruitfly.org/cgi-bin/ex/insitu.pl) [23]. It was subsequently shown by immunolocalization that DSX expression in the central nervous system is restricted to a small subset of neurons in adults [24]–[26] and that DSX^M^ expression in male gonads is restricted to somatic cells in both embryos and adults [27]. However, characterization of the spatial and temporal expression pattern of *dsx* has been quite limited. Moreover, the implications of a restricted pattern of *dsx* expression have been minimally considered.

To better understand the function of *dsx* in sexual development and the sexuality of adults, we generated a tool with which we could both visualize *dsx* expression throughout development and also manipulate *dsx*-expressing cells. Specifically, we used homologous recombination to insert the *GAL4* transcriptional activator coding sequence into the *dsx* locus immediately following the start codon to generate *dsx^GAL4^*. In only those cells that express it, *dsx^GAL4^* can be used to drive the expression of any transgene of interest under control of the GAL4-responsive upstream activating sequence (UAS). *dsx^GAL4^* faithfully reproduces the known features of *dsx* expression. In addition, we observed *dsx^GAL4^* expression in many tissues where no phenotypic effects of *dsx* mutants have been reported. Strikingly, *dsx*, like *fru*, is only expressed in a subset of tissues and is highly restricted within those domains; in many cells and tissues, it is not expressed at all. Thus in both *XY* and *XX* Drosophila, only a subset of cells appear to have the potential to sexually differentiate, and hence both males and females are mosaics of sexually differentiated and sexually undifferentiated cells. These findings have led to a significant revision in our understanding of how sex is specified in Drosophila. These findings also have significant implications for evolutionary considerations of sex.

## Results

### Targeting *GAL4* Coding Sequence into the *dsx* Locus

Alternatively spliced mRNAs derived from a common promoter and common 5′ exons encode the DSX^M^ and DSX^F^ proteins, which have the same N-terminus but different C-termini [28]. We used homologous recombination to insert the *GAL4* coding sequence immediately after the translational start codon of the *dsx* gene in exon 2, which is common to male and female *dsx* mRNAs, to generate *dsx^GAL4^* (Figure 1B) [29]. The *GAL4* coding sequence is terminated by a single stop codon. The two codons 3′ of the *GAL4* stop codon encode different amino acids than those found in the DSX proteins, but otherwise the *dsx* sequences both 5′ and 3′ of the *GAL4* insertion site are unaltered in the targeted chromosome. Thus, the *dsx^GAL4^* gene is anticipated, barring internal reinitiation of translation, to be a null allele of *dsx*.

The proper targeting of the *GAL4* coding sequence into the *dsx* gene was confirmed by genomic PCR. Additionally, we found that the insertion creates a mutant allele of *dsx* that produces classical *dsx* morphological phenotypes when heterozygous with the null alleles *dsx^1^* or *In(3LR)dsx^M+R13^* (unpublished data). However, the *dsx^GAL4^* chromosome unexpectedly causes reduced fertility in both sexes.

### Validation of the Expression Pattern of *dsx^GAL4^*

Both molecular and genetic characterizations of the *dsx^GAL4^* expression pattern indicate that it faithfully recapitulates the endogenous *dsx* expression pattern.

**Figure 2:**
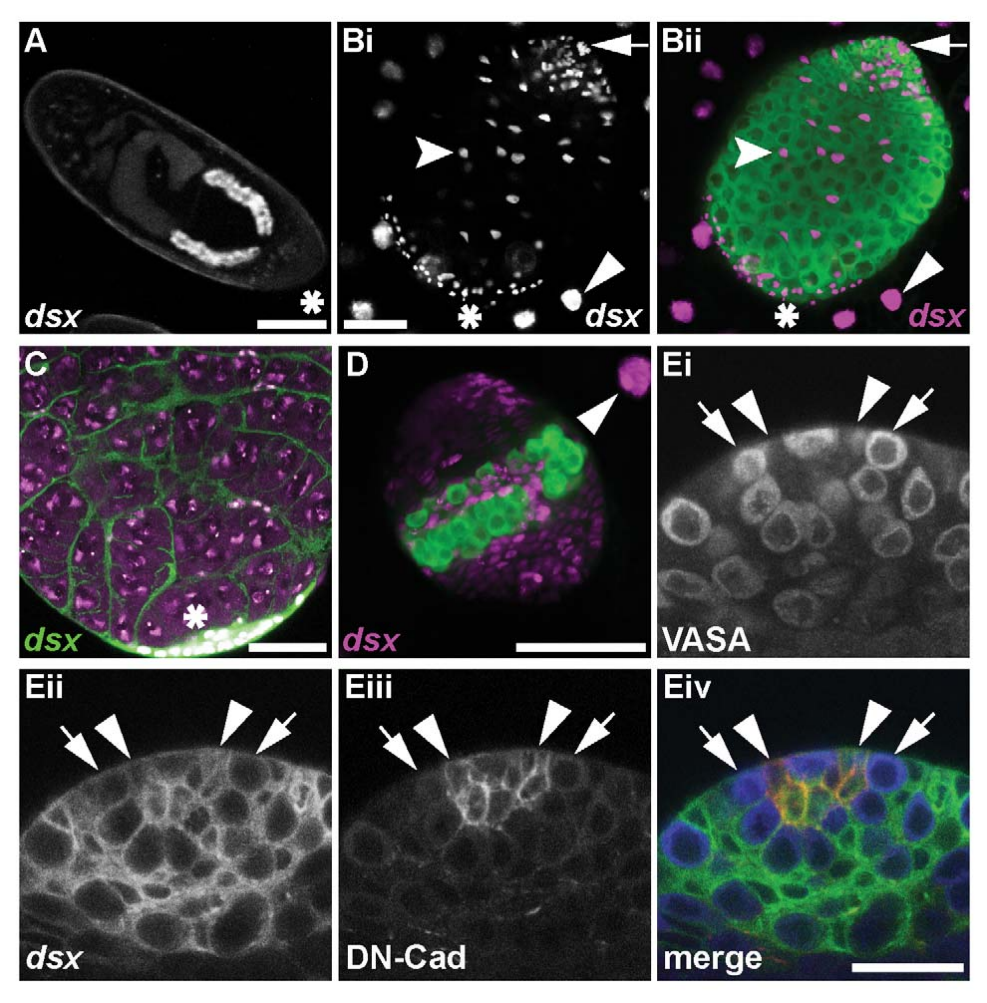
*dsx^GAL4^* driving UAS-induced fluorescent reporters recapitulates known patterns of gonadal *dsx* expression. (A) *dsx^GAL4^* expression is restricted to cells of the paired uncoalesced gonads in embryonic stage 13. The embryo posterior is marked by an asterisk, and native GFP fluorescence was imaged from the membrane reporter, *UAS-mCD8::GFP*. Scale bar, 100 µm. (B–C) Expression of *dsx^GAL4^* in the male third instar larval gonad is restricted to somatic cells. Reporter is *UAS-RedStinger* (nuclear DsRed). (Bi) *dsx^GAL4^*-expressing cells include the clustered hub cells of the stem cell niche at the gonad apical tip (arrow), cyst cells dispersed throughout the gonad (barbed arrowhead), and terminal epithelial cells with small round nuclei at the basal end of the gonad (asterisk). A fat body cell nucleus is indicated (arrowhead). (Bii) *dsx^GAL4^* expression (magenta) is excluded from the germline cells marked with cytoplasmic VASA (green). Scale bar, 50 µm. (C) Expression of the *JFRC-IVSA* membrane-bound GFP reporter (green) reveals sheath cell cytoplasmic processes ramifying through cysts of male germ cells in the basal half of the gonad. DNA of the large male germline cells is stained with DAPI (magenta). Gonad posterior indicated with an asterisk. Single confocal section shown. Scale bar, 20 µm. (D) *dsx^GAL4^* expression in the female third instar larval gonad is restricted to somatic cells (magenta) and is excluded from the germline cells marked with cytoplasmic VASA (green). A fat body cell nucleus is indicated (arrowhead). Reporter is *UAS-RedStinger* (nuclear DsRed). Scale bar, 50 µm. (E) *dsx^GAL4^* expression in hub cells of the stem cell niche at the apical tip of male the gonad was visualized with the *JFRC-IVSA* membrane-bound GFP reporter (Eii; green in Eiv). Processes of *dsx^GAL4^*-expressing cells enveloped germline cells marked with VASA (Ei; blue in Eiv), including the ring of germline stem cells adjacent to the hub (arrows). Expression in hub cells is confirmed by overlap with DN-Cadherin (DN-Cad) (Eiii; red in Eiv), which marks membranes of the hub cells (arrowheads). Overlap of membrane-bound GFP with DN-Cadherin is yellow in the merge (Eiv). Scale bar, 50 µm.

*dsx* transcripts and DSX^M^ protein are only detected in cells of the developing gonads in stage 13–17 embryos (www.fruitfly.org/cgi-bin/ex/insitu.pl) [23],[27], and we saw *dsx^GAL4^* driven *UAS-mCD8::GFP* [30] expression that precisely recapitulated this expression pattern (Figure 2A). This pattern of reporter gene expression was completely dependent on the presence of *dsx^GAL4^*, as were all other patterns of *dsx^GAL4^* expression described below.

In third instar larvae, *dsx^GAL4^* was expressed in the gonads of both sexes (Figure 2B,D), consistent with detection of DSX^M^ at these stages [27]. Further, *dsx^GAL4^* expression is coincident with the protein EYES ABSENT (EYA), which marks all somatic cell nuclei of the gonad (unpublished data) [31], but *dsx^GAL4^* was not expressed in germ cells, which are marked by the cytoplasmic protein VASA (Figure 2B,D) [32]. Somatic cells in which *dsx^GAL4^* was evident include the apical hub cells that form the germline stem cell niche (Figure 2E), cyst cells that envelop the developing germ cells throughout spermatogenesis (Figure 2B,C), and cells at the basal end of the testis that likely correspond to male-specific gonadal precursor cells (Figure 2B,C) [33]. This pattern of expression, like that of DSX^M^, continued into the adult testis. Thus, *dsx^GAL4^* expression recapitulates the DSX^M^ pattern in the gonad and reveals expression in the female gonad.

DSX antibodies have also been used to describe *dsx* expression in the CNS [24]–[26]. As presented below, *dsx^GAL4^* expression in the CNS recapitulates all major features of the reported temporal and spatial patterns of DSX expression and in addition reveals new features of *dsx* expression in the CNS.

**Figure 3:**
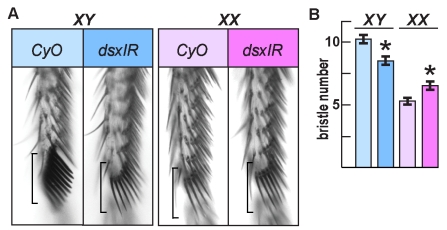
*dsx^GAL4^*-induced expression of *UAS-dsxIR* at 29°C causes transformation of a sexually dimorphic row of leg bristles toward an intersexual morphology. (A) Tarsal segment 1 from chromosomal males (*XY*) and females (*XX*) with the *dsx^GAL4^* chromosome alone (*CyO*, balancer chromosome) or driving *UAS-dsxIR*. The regions of the male sex comb and homologous female last transverse bristle row are bracketed. *UAS-dsxIR* causes the thickness, pigmentation, and taper of bristles to look the same in males and females. (B) Quantitation of bristle numbers from experiment in (A). Histogram bars for the *UAS-dsxIR* condition are marked with an asterisk for each chromosomal sex. *XY CyO* males (*n* = 17 legs) have an average of 10.2±0.62 (standard deviation) bristles, which is reduced to 8.5±0.61 in *XY UAS-dsxIR* (*n* = 19). *XX CyO* females (*n* = 19) have an average of 5.35±0.49 bristles, which is increased to 6.47±0.611 in *XX UAS-dsxIR* (*n* = 18).

To further assess *dsx^GAL4^*'s accuracy, we asked if it was expressed in cells that give rise to the sexually dimorphic external parts of the fly. The major external sexual dimorphisms in Drosophila occur on the forelegs, tergites (dorsal aspects of abdominal segments), sternites (ventral aspects of abdominal segments), and the genitalia. In all of these regions, males and females differ in the number, location, morphology, and/or pigmentation of specific cuticular elements. To ascertain whether *dsx^GAL4^* was expressed in the cells producing these sexually dimorphic cuticular elements, we asked whether *dsx^GAL4^*-directed expression of an inhibitory RNA (*UAS-dsxIR*) that targets both the wild-type male and female *dsx* transcripts would produce *dsx* mutant cuticular phenotypes. Intersexual differentiation was seen in all of these cuticular elements in *dsx^GAL4^/dsx^+^* individuals with two copies of *UAS-dsxIR* reared at 29°C, although to a lesser degree in some cases than what was displayed by *dsx* null control individuals, possibly indicating that *UAS*-*dsxIR* did not fully suppress *dsx^+^* expression and/or also targeted the fused *GAL4-dsx* transcripts. For example, on the first tarsal segment of forelegs of wild-type individuals, there are *ca.* 10 bristles that develop as the thick, blunt sex comb teeth in the male (Figure 3A, *XY*) and *ca.* 5 tapered, pointed homologous bristles in the female (Figure 3A, *XX*) [34],[35]. In both *XY* and *XX dsx* mutant individuals, these bristles are intermediate in both their number and morphology between those of wild-type males and females. In both *XY* and *XX* individuals in which *dsx^GAL4^*/+ drove expression of *UAS-dsxIR*, these bristles had an intermediate morphology (Figure 3A). However, the sexual dimorphism in bristle number was not eliminated by *UAS*-*dsxIR* expression, although the numbers of these bristles were significantly decreased in males and increased in females (Figure 3B). Differentiation of non-sexually dimorphic regions of the cuticle was normal in both sexes (unpublished data). Taken together, the above findings are all consistent with *dsx^GAL4^* accurately reporting the *dsx* expression pattern.

Below, we extended these studies by examining *dsx^GAL4^*-driven expression of UAS-fluorescent protein reporters at many developmental stages. Except as noted for sensory organs of the foreleg and genitalia, and the CNS, we saw no difference between males and females in the *dsx^GAL4^* expression patterns and our descriptions apply to both sexes. The results reveal a very dynamic and elaborate pattern of *dsx^GAL4^* expression in a wide variety of cell types reflecting the transcriptional regulation of *dsx* across development.

### *dsx* Expression in Embryos, and First Instar and Second Instar Larvae

In embryos, *dsx^GAL4^* expression was only detected in the gonad, as described above. We did not see expression in the genital imaginal disc precursor cells [36]. In first instar larvae, expression was seen in *ca.* 4 cells that are likely part of the genital disc based on their location (unpublished data). The number of genital disc cells expressing *dsx^GAL4^* increased in the second instar to *ca.* 8–10 cells, which is substantially below the number of cells in the genital disc in young larvae (*ca.* 60) as determined by direct counts [37].

### *dsx* Expression in Third Instar Larvae

In intact third instar larvae, fluorescence from *dsx^GAL4^*-driven reporters in the gonads and genital disc was easily detectable through the body wall. Fluorescence from other discs, some of which do express *dsx^GAL4^* (see below), was not routinely detectable in intact larvae. In very late stage third instar larvae (wandering stage), *dsx^GAL4^* expression became detectable in most, if not all, of the larval fat body cells of both sexes (unpublished data). Larval fat body expression of *dsx^GAL4^* was detected with three different UAS-fluorescent protein reporters and was never seen in the absence of *dsx^GAL4^* and represents the first case we know of in *D. melanogaster* where a purely larval tissue may be sexually dimorphic.

### *dsx* Expression in Third Instar Larval Imaginal Discs

**Figure 4:**
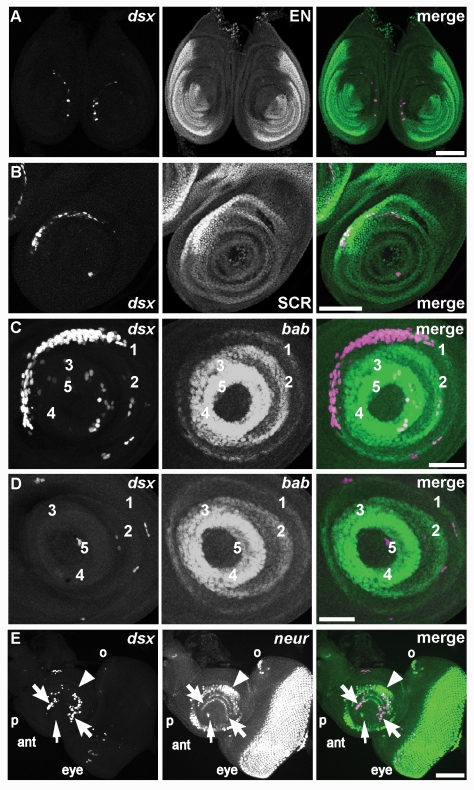
*dsx^GAL4^* is expressed in imaginal discs. Fluorescence of indicated reporters in imaginal discs of third instar female larvae is shown. (A) *dsx^GAL4^* driving expression of *UAS-Stinger* nuclear GFP reporter (magenta) is compared to the posterior compartment of the disc marked by EN (green) in this pair of foreleg discs from an early wandering third instar larva. At this stage, few cells express *dsx^GAL4^*, but the majority of these cells form a broken crescent within the anterior compartment. Scale bar, 100 µm. (B) Expression of *UAS-Stinger* nuclear GFP reporter (magenta) in the foreleg disc is compared to the position of tarsal and tibial primordia that express high levels of SCR (green). In this disc from a more mature wandering third instar larva than shown in (A) there are more expressing cells in the *dsx^GAL4^* crescent, and the crescent overlaps with the SCR domain. Scale bar, 100 µm. (C) Expression of *UAS-RedStinger* nuclear DsRed reporter (magenta) is compared to the position of tarsal segment boundaries defined by concentric expression of *bab-lacZ* (green). In this foreleg disc from a third instar larva shortly before purarium formation, there is a greater number of cells expressing *dsx^GAL4^* in the *dsx^GAL4^* crescent, and the crescent overlaps with the *bab-lacZ* domain corresponding to the T1 tarsal segment. Scale bar, 50 µm. (D) Expression of *UAS-RedStinger* nuclear DsRed reporter (magenta) in a leg disc corresponding to the second or third leg is compared to the position of *bab-lacZ* (green). In contrast to (A–C), there are only a few cells expressing *dsx^GAL4^* scattered across the disc, and there is no crescent. Scale bar, 50 µm. (E) Expression of *UAS-RedStinger* nuclear DsRed reporter (magenta) is compared to the position of cells expressing the proneural marker *neur-lacZ* (green) in the eye-antennal disc from a mature wandering third instar larva. Many cells express *dsx^GAL4^* in the antennal (ant) portion of the disc, while only a few cells express *dsx^GAL4^* in the eye portion. Primordia for the second (arrowhead) and third (barbed arrows) antennal segments are indicated, as are primordia for the arista (arrow), palpus (p), and ocellus (o). Scale bar, 50 µm.

Since *dsx* is known from genetic data to function during development in several imaginal discs [34],[35],[38],[39], we examined *dsx^GAL4^* expression in dissected imaginal discs of mature third instar larvae using the nuclear reporter *UAS-RedStinger*. No *dsx^GAL4^* expression was seen in clypeolabral, labial, or humeral discs. In the second leg, third leg, wing, and haltere discs there were only a few cells expressing *dsx^GAL4^*, mostly in the region near the stalks of these discs, and the number of *dsx^GAL4^*-expressing cells increased with age so that at the time of puparium formation the number of labeled cells in the wing disc approached 30, the haltere 20, and the second and third legs 5–15 each (Figure S1; see Figure 4E). The nature of these cells and what parts of the adult they correspond to is unknown. There are no known anatomic sexual dimorphisms in the adult derivatives of these discs. In the genital, foreleg, and eye-antennal discs, some of whose adult derivatives are sexually dimorphic, there was significant *dsx^GAL4^* expression.

In the genital disc of both sexes, *dsx^GAL4^* was broadly expressed, consistent with the fact that all major regions of this disc give rise to sexually dimorphic structures of the adult genitalia and analia [2],[40]. The only regions of the genital disc not expressing GFP were the lateral edges of the disc (Figure S1).

In contrast, foreleg discs from early wandering third instar larvae exhibited *dsx^GAL4^* expression in a thin, crescent-shaped band of epithelial cells plus several scattered cells in both sexes (Figure 4A). The crescent of foreleg disc cells expressing the highest levels of fluorescence is outside of the *engrailed* domain of the disc, indicating that these cells reside in the anterior compartment (Figure 4A). However, a few cells with lower levels of fluorescence could be seen scattered within the *engrailed* domain. At this stage, a total of 15–30 cells expressed *dsx^GAL4^* in the foreleg disc.

In mature wandering third instar larvae, the crescent of *dsx^GAL4^*-expressing cells in the foreleg disc increased markedly. *dsx^GAL4^* expression largely overlapped with *Sex combs reduced* protein (SCR) expression, consistent with the known requirement for both *Scr* and *dsx* in sex comb formation (Figure 3B) [35],[41]. The relative levels of *dsx^GAL4^* and SCR expression varied significantly between cells. Further examination of foreleg discs from less mature third instar larvae showed that a low level of SCR could be detected in all tarsal regions of the disc at a time when *dsx^GAL4^* was expressed in only a few cells (unpublished data), suggesting that the specification of tarsal segment identities by *Scr* precedes expression of *dsx^GAL4^*. The crescent of *dsx^GAL4^* expression was in tarsal segment 1 (T1) based on its location relative to that of *bric-a-brac-lacZ* (*bab-lacZ*) (Figure 4C), which labels the tarsal segment boundaries [42].

In contrast to the foreleg disc, *dsx^GAL4^* was expressed in only a few cells within the tarsal segments of the other leg discs in mature third instar larvae (Figure 4D). Thus, the pattern of *dsx^GAL4^* expression in the foreleg disc epithelium is dynamic during late larval life.

*dsx^GAL4^* was expressed in subsets of cells within both portions of the eye-antennal disc (Figure 3E). In the eye domain, there were *ca.* 32 such cells with the majority residing in the ventral region of the disc. Although a number of these cells were within or immediately below the plane of the photoreceptor cells, only a few of them expressed the proneural marker *neuralized-lacZ* (*neur-lacZ*) [43]. An additional *ca.* 10 *dsx^GAL4^*-expressing cells were in the region of the frons primordia, while additional cells having very faint expression were found in both eye disc regions. In the antennal domain of the eye-antennal disc of mature wandering third instar larvae, there were *ca.* 71 cells expressing *dsx^GAL4^*. Approximately 10 of these were found in segment 1, located near the stalk opposite the palpus, and the remaining *ca*. 61 were found in segment 3, which contains the olfactory sensory organ precursors, and segment 4. Of the cells expressing *dsx^GAL4^* in segments 3 and 4, a small number co-expressed *neur-lacZ*, suggesting they may be sensory organ precursors. No *dsx^GAL4^* expression was seen in segment 2, the Johnston's organ primordia; segment 5, the basal cylinder primordia; or segment 6, the arista primordia. Like the thoracic discs, the number of cells expressing *dsx* in the eye-antennal disc undergoes a dramatic increase over the course of late larval life.

### *dsx* Expression in Other Internal Larval Tissue

We examined other imaginal tissues of mature wandering third instar larvae for *dsx^GAL4^* expression. In the gut, expression was detected in a number of small cells in a region posterior to the slight tapering of the anterior midgut (unpublished data). Each of these cells localized to clusters of cells that constitute the gut imaginal nests, and they were thus imaginal cells fated to contribute to the adult midgut. The number of such cells expressing *dsx^GAL4^* ranged between 12 and 17 in males and 4 and 7 in females, but this difference may reflect variability in the degree of maturity of the small number of larvae examined. Notably, in those imaginal nests containing at least one *dsx^GAL4^*-expressing cell, the majority of other cells in the cluster did not express *dsx^GAL4^*. Further, other gut imaginal nests in the anterior midgut and those in posterior regions of the gut did not express *dsx^GAL4^* at the stage examined. Nor was expression observed in the tracheal nests, abdominal histoblast nests, gut imaginal rings, or salivary gland imaginal rings.

### *dsx* Expression in Adult Tissues

*dsx^GAL4^* was expressed in the cells of most (but not all) adult tissues in which *dsx* is known to have a developmental role. Expression in adult adipose cells (or fat body) and oenocytes was anticipated from genetic studies [2],[44], and we observed *dsx^GAL4^* expression in the large sheets of adipose cells associated with the dorsal abdominal body wall, as well as in oenocytes (Figure S2). *dsx^GAL4^* was also expressed in all of the internal derivatives of the genital disc in both sexes with the notable exception of the male accessory gland, which showed no expression (unpublished data). With respect to the lack of adult expression of *dsx^GAL4^* in the male accessory gland, it is worth noting that *dsx* function is required during the late larval period, but not subsequently, for its specification [45].

Muscles that attach to sex-specific elements of the internal and external genitalia, such as the ejaculatory bulb and penis apparatus in males and the gonopod in females, also express *dsx^GAL4^* (Figure S5 and unpublished data), as do epidermal cells underlying the cuticular elements of the external male genitalia and analia (Figure 6C,D). Consistent with *dsx*'s regulation of cuticle pigmentation in abdominal tergites 5 and 6 [2], *dsx^GAL4^* was expressed in many epidermal cells underlying the abdominal cuticle (unpublished data). In the five tarsal segments of the foreleg, *dsx^GAL4^* was expressed in a complex pattern as outlined below.

**Figure 5:**
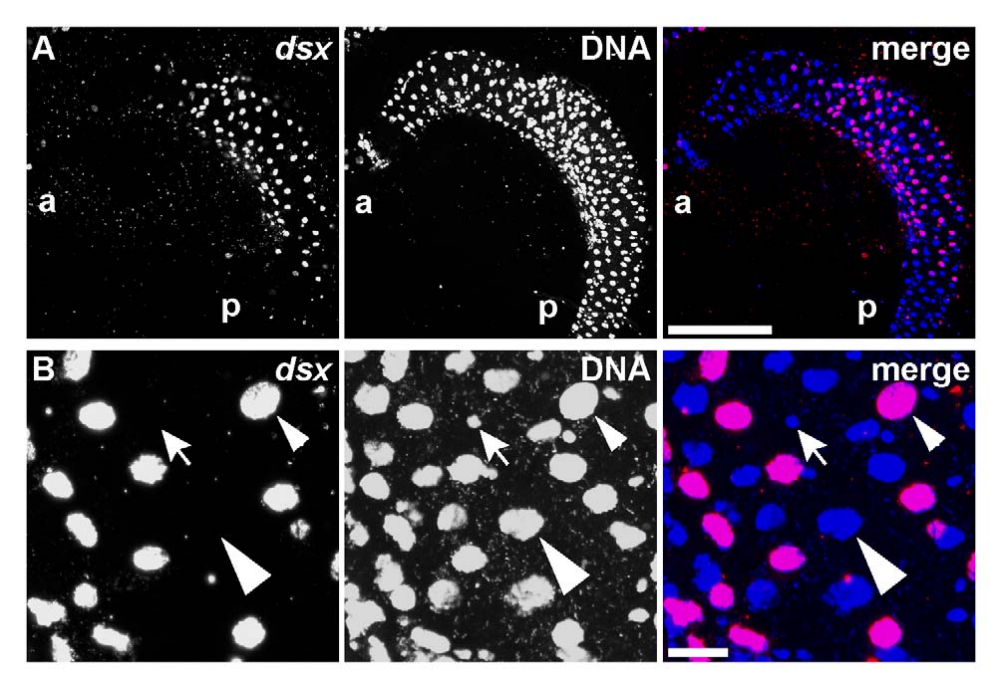
*dsx^GAL4^* is expressed in a subset of cells of the midgut. (A) The frequency of cells expressing *UAS-RedStinger* nuclear DsRed reporter (magenta) varies along the length of the anterior midgut from an adult female. Non-expressing nuclei are marked with DAPI alone (blue). Anterior (a) and posterior (p). Speckles of autofluorescent debris are seen in the area outside of the gut. Scale bar, 100 µm. (B) Magnified view of a region in (A). Some enterocytes express *dsx^GAL4^* (small arrowhead) while others do not (large arrowhead). In this region of the gut, cells with smaller nuclei (barbed arrow) that are likely to be enterocrine or intestinal stem cells do not express *dsx^GAL4^*. Scale bar, 10 µm.

Intriguingly, *dsx^GAL4^* was also expressed in a number of locations and cell types for which there are no reported sexual dimorphisms, including thoracic and leg muscles, various head structures, epidermal cells, and subsets of cells in the digestive system. *dsx^GAL4^* was also expressed in a subset of tracheal cells of the abdomen, although consecutive cells along the same tracheole varied between expressing or not expressing (unpublished data). Expression was also seen in trachea associated with the internal genitalia and the gonads. *dsx^GAL4^* was also expressed in cells of the Malpighian tubules and salivary glands (Figure S3 and unpublished data). While we did not examine all muscles, we observed expression in muscle groups of the proboscis, the tergosternal muscles underlying the ventral abdominal cuticle, many of the muscles that overlay the proventriculus and midgut (unpublished data), as well as muscles associated with parts of the reproductive structures mentioned above. In addition, *dsx^GAL4^* was expressed in muscles of the mesothorax, as well as the trochanter, coxa, femur, and tibia of all three pairs of legs (Figure S4 and unpublished data).

*dsx^GAL4^* expression in the digestive system illustrates the complex transcriptional regulation governing *dsx* expression. *dsx^GAL4^* was expressed in the proventriculus, midgut, rectum, and crop of the adult digestive system (Figure S3). In each of these components, expression is spatially restricted to subsets of cells, as revealed by both nuclear and membrane reporters (*UAS-RedStinger* and *UAS-mCD8::GFP*, respectively). In the proventriculus, *dsx^GAL4^* was expressed in subsets of epithelial cells distributed across the epithelial folds of this organ and was expressed in much of the epithelium of the stomodaeal valve (Figure S3) [46]. Great variability in *dsx^GAL4^* expression was seen in the populations of large enterocytes residing in different regions along the length of the midgut (Figure 5A,B). Smaller nuclei likely corresponding to both the basally located intestinal stem cells and the enteroendocrine cells [47],[48] did not express *dsx^GAL4^* in the regions examined. In contrast to the midgut and rectum, no expression was detected in cells of the hindgut that connects them (Figure S3 and unpublished data). While *dsx^GAL4^* was expressed in many cells covering the rounded surface of the rectum, it was not expressed in the large epithelial cells forming the rectal papillae (epithelial folds) that project into the lumen of the rectum (unpublished data). Expression was also absent in cells forming the tracheoles that extend into each rectal papilla.

### *dsx* Expression in the Peripheral Nervous System

While *dsx* is clearly implicated in distinct aspects of sexual behaviors [26],[49] and has been shown to have roles generating sexual dimorphisms in both peripheral [50] and central neurons [24],[26],[51],[52], it has been difficult in most cases to link *dsx*-dependent behavioral deficits to particular aspects of the nervous system (but see [26],[51]). Part of the difficulty has stemmed from the fact that most courtship defects detected in *dsx* null males manifest as general decrements in courtship. These general decrements in courtship could reflect requirements for *dsx* in the CNS, peripheral sensory neurons, or even non-neuronal cells.

As a first step towards distinguishing between these possibilities, we have characterized in detail the patterns of *dsx^GAL4^* expression in the second and third antennal segments, tarsal segments of the foreleg, proboscis, maxillary palp, and external genital structures, which collectively contain primary sensory neurons of the olfactory, gustatory, auditory, and mechanosensory systems that are known to be important for courtship. In those instances where *dsx^GAL4^* was expressed in peripheral sensory structures known to contain *fru*-expressing neurons, we examined whether their expression overlaps at the cellular level. To do this, we simultaneously imaged expression of *dsx^GAL4^* and *fru^P1.LexA^* [50] using UAS-nuclear GFP (*UAS-Stinger*) and *lexA operator-nuclear tdTomato* (*lexAop-tdTomato::nls*) red fluorescent protein [50] in males and females at 72 h after puparium formation (APF) and as 0–12 h adults. The patterns of *dsx^GAL4^* expression seen at these two time points were virtually the same.

**Figure 6:**
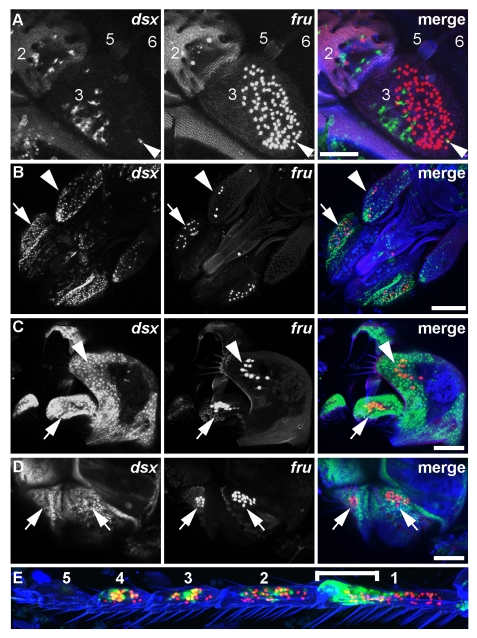
*dsx^GAL4^* and *fru^P1.LexA^* expression in peripheral sensory structures. Expression of *dsx^GAL4^* and *fru^P1.LexA^* at 72 h APF. In the *fru^P1.LexA^* chromosome, LexA::VP16 coding sequence followed by the SV40 poly-A signal and α-tubulin 3′UTR sequences as transcriptional termination sequences were inserted into the *fru* gene [50]. *dsx^GAL4^* was detected by expression of *UAS-Stinger* nuclear GFP reporter (green), and *fru^P1.LexA^* was detected by expression of *lexAop-tdTomato::nls* (red). Cuticle autofluorescence is in blue. Confocal Z projections are shown. (A) In the antenna, *dsx^GAL4^* is expressed in a subset of cells that are largely distinct from the population of neurons expressing *fru^P1.LexA^*. In segment 2, cells near the base of the large mechanosensory bristles express *dsx^GAL4^*. In segment 3, the majority of cells expressing *dsx^GAL4^* lie close to the cuticle and do not have a neuronal morphology. Neurons expressing both genes (yellow) were rarely observed (arrowhead). Female antenna shown. Scale bar, 50 µm. (B) In the proboscis and maxillary palps, no cells express both *dsx^GAL4^* and *fru^P1.LexA^*. (Nonspecific overlap is artifact of Z projection.) At the tip of the maxillary palp (arrowhead), *dsx^GAL4^* is expressed in cells near the base of the large mechanosensory bristles. In the maxillary palps and the proboscis (arrow), there are many pericuticular *dsx^GAL4^*-expressing cells. Female structures shown. Scale bar, 100 µm. (C) In the male external genitalia, *dsx^GAL4^* is broadly expressed in pericuticular cells, as well as in neurons of the claspers (arrow), which also express *fru^P1.LexA^*. *dsx^GAL4^* is not expressed with *fru^P1.LexA^* in neurons of the lateral plates (arrowhead). The genitalia are viewed from a side angle, and anterior is up. Scale bar, 50 µm. (D) In the paired male anal plates, *dsx^GAL4^* is broadly expressed in pericuticular cells (arrows) but is not co-expressed with *fru^P1.LexA^* in mechanosensory neurons. The analia lie posterior to the genitalia and are viewed from a side angle. Scale bar, 50 µm. (E) Tarsal segments T1–T5 of the male foreleg. The tarsal segments are numbered 1–5, proximal to distal. Expression of *dsx^GAL4^* is seen in a subset of *fru^P1.LexA^*-expressing neurons associated with gustatory sense organs, most visible in segments 3 and 4. Pericuticular expression is seen in the region of the sex comb (bracket). (Overlap in tarsal segment 5 is not visible in this projection.)

In the head, *dsx^GAL4^* was not expressed in any of the chemosensory or auditory neurons of the antennae, maxillary palps, or proboscis; accordingly, there is no overlap with *fru^P1.LexA^*. We did observe expression in other, non-neuronal cells in these tissues. For example, *dsx^GAL4^*-expressing cells were associated with the bases of large mechanosensory bristles on the second antennal segment and maxillary palps, and *dsx^GAL4^* was expressed in epithelial cells along the lateral aspect of the proboscis (Figure 6A,B). Several days after eclosion, we also observed expression of *dsx^GAL4^* in cells of the basal cylinder, the small antennal segment from which the arista projects (unpublished data).

At the late pupal/young adult stage, *dsx^GAL4^* was expressed in a complex pattern in the five tarsal segments of the foreleg. In all five tarsal segments, *dsx^GAL4^* was expressed in a subset of gustatory sense organs (GSOs), as evidenced by its pattern of expression with respect to that of *fru^P1.LexA^*, which is expressed in all foreleg GSOs with the exception of two GSOs in tarsal segment 5 (Figure 6E) [50]. Each GSO of the foreleg is composed of four gustatory neurons, one mechanosensory neuron, and several non-neuronal cells [53]. In those GSOs in which it was expressed, *dsx^GAL4^* was expressed in both neuronal and non-neuronal cells (unpublished data). In tarsal segments 1–4 *dsx^GAL4^* was expressed in fewer GSOs in females than in males (unpublished data), likely because males have more foreleg GSOs than do females [53]. In the first tarsal segment, *dsx^GAL4^* was expressed in cells associated with the sex comb bristles, as well as pericuticular cells around the sex comb. Less pericuticular expression was seen in other tarsal segments.

*dsx^GAL4^* was co-expressed with *fru^P1.LexA^* in the neurons of the clasper bristles of the male genitalia, but not in *fru^P1.LexA^*-expressing neurons of the mechanosensory bristles of the lateral plate and anal plate (Figure 6C,D).

### *dsx* Expression in the CNS

**Figure 7:**
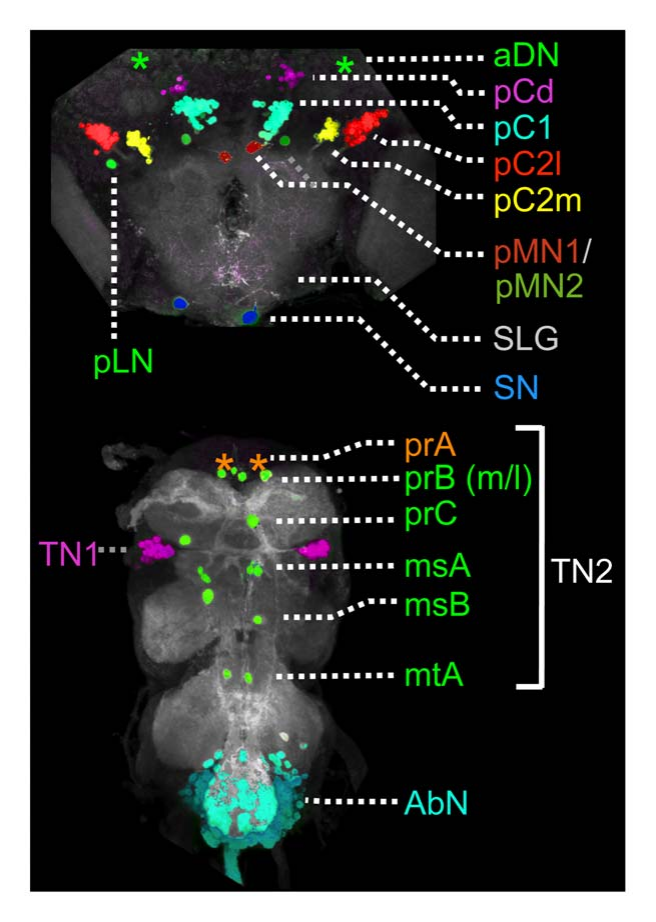
Diagram of *dsx^GAL4^* expression in the CNS. Identity of neurons and neuron clusters expressing *dsx^GAL4^* in the male brain and VNC are diagrammed following the nomenclature of Lee et al. [26] with several additions. Positions of neurons not visible in this projection are represented by asterisks based on their position in other samples. In the brain, we observed the following previously identified neurons: anterior dorsal neurons (aDN, not visible in this projection), posterior clusters pC1 and pC2, and the suboesophageal neurons (SN). We subdivide pC2 into lateral and medial clusters (pC2l and pC2m, respectively) based on their projections. We add the posterior dorsal cluster (pCd) and identify two distinctive posterior Medial Neurons (pMN1 and pMN2) near cluster pC1, as well as one isolated posterior Lateral Neuron (pLN) within each hemibrain. The pMN neurons are also identifiable in females. We did not observe the Suboesophageal Lateral Neurons (SLNs) reported by Lee et al. [26] but instead note a dispersed population of putative glia in the anteroventral optic cleft (see Figure 8). In the VNC, we observe the previously identified prothoracic TN1 neurons and several single and paired thoracic TN2 neurons. We distinguish the latter by their segmental identity as prothoracic (pr), mesothoracic (ms), or metathoracic (mt) TN2 neurons; prA (not visible in this sample) resides on the dorsal cortex of the VNC, while the rest are found ventrally. We also distinguish the prB TN2 neurons as medial (prBm) and lateral (prBl) neurons. The abdominal cluster (AbN) is also visible in both males and females.

We visualized *dsx^GAL4^* expression in the CNS using the *UAS-mCD8::GFP* membrane-bound GFP reporter and observed excellent correspondence with the *dsx* neuron clusters previously identified by immunolocalization (Figure 7) [26]. We observed prominent expression of *dsx^GAL4^* in neurons of the posterior brain, including the pC1 and pC2 clusters. Both were sexually dimorphic, with females showing ∼85% fewer cells in the medial pC1 cluster and ∼80% fewer cells in the pC2 cluster relative to males (Figure 7). The pC2 cluster also appeared to consist of at least two distinct clusters, which were each associated with distinct fasciculations from the medial (pC2m) and lateral (pC2l) clusters.

We also noted a small cluster of *dsx^GAL4^*-expressing neurons in the dorsal aspect of the posterior brain that had not been reported previously, which we here identify as posterior Cells, dorsal (pCd) (Figure 7). This cluster is apparent at mid-pupal stages in previous work, but it was not named (Figure 4 in [26]; Figure 1 in [25]; Figure S1 in [26]). Females had ∼50% fewer neurons in this cluster than did males. We also confirmed expression in a small number of isolated neurons in the male brain, including two anterior-dorsal neurons (aDNs), and a single medial suboesophageal neuron (SN) within each hemibrain; these neurons were absent in females (Figure 7). In addition to these clusters, we noted several neurons that had been previously identified as being part of the pC1 and pC2 clusters, but their distinct fasciculations and large cell bodies suggested that they are unlikely to be associated with these clusters. We rename these neurons in Figure 7.

**Figure 8:**
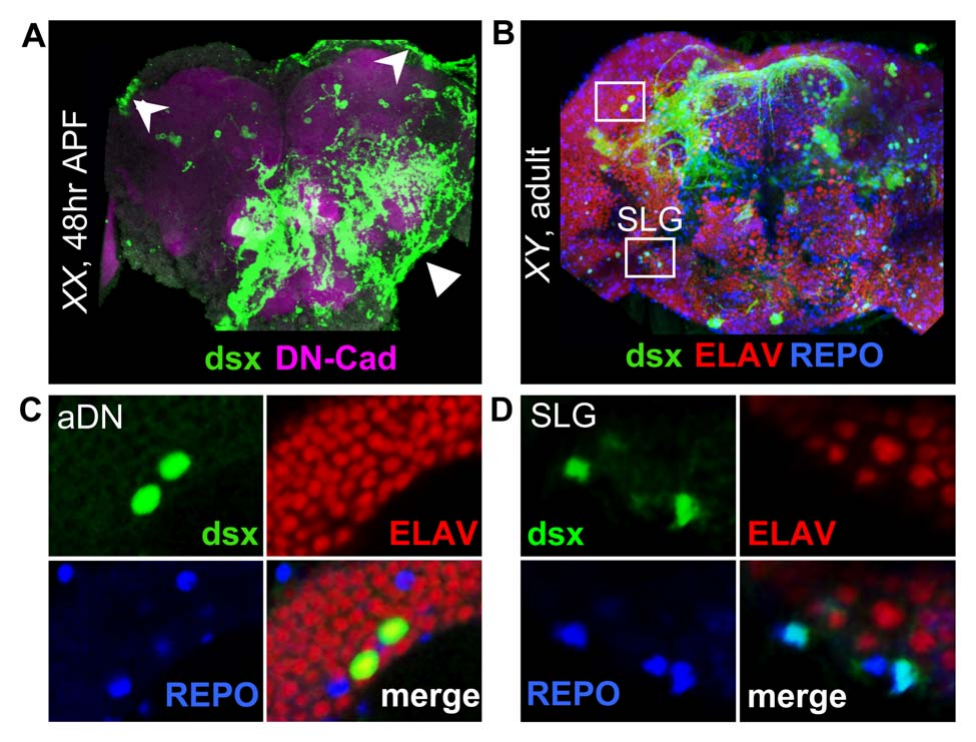
*dsx^GAL4^* is expressed in a subset of glial cells in the CNS. (A) *dsx^GAL4^* is expressed in cells with glial morphology (arrows) that are associated with the cortical surface of the brain in a female at 48 h APF, as shown using *UAS-mCD8::GFP* membrane-bound GFP reporter. Shown here is a projection of the anterior brain, revealing cells across the medial optic cleft and suboesophageal ganglion (arrowhead). Expression is also seen in some samples at the cortical surface of the lateral horn (barbed arrowheads). Neuropile is counterstained with DN-Cadherin (DN-Cad). (B) *dsx^GAL4^* expression in the anterior brain of a 0 d adult male using the *UAS-Stinger* nuclear GFP reporter. Costaining for the neuronal marker ELAV (red) and the glial marker REPO (blue) reveals overlap with *dsx^GAL4^* in a subset of neurons and glia. In the region of the anterior Dorsal Neurons (aDN, detailed in panel C), we observe overlap with ELAV but not REPO. However, in the anteroventral optic cleft, we observe overlap with REPO but not ELAV, confirming the glial cell type of the Suboesophageal Lateral Glia (SLG; detailed in panel D). (C) The aDNs (green) are positive for ELAV (red) but not REPO (blue). (D) The SLG in the anteroventral optic cleft are positive for REPO but not ELAV.

Although previous authors have reported a neuronal morphology for all *dsx^GAL4^*-expressing cells in the anterior brain, we could not confirm this for the suboesophageal lateral neurons (SLNs), located in the anteroventral optic cleft [25],[26]. In this area, we instead observed a novel pattern of expression in gliaform cells (Figure 8A,B). These putative glia had a cortical morphology and were seen in both sexes beginning at about 24 h APF (unpublished data). Although they first appeared in the ventrolateral optic cleft, we observed a scattered distribution of these cells at later time points across the anterolateral cortex of the suboesophageal ganglion (SOG), consistent with the migratory behavior of glia during CNS development (review [54]). We confirmed these cells to be glia by examining overlap with the glial marker REPO (Figure 8B,D), and we refer to them as Suboesophageal Lateral Glia (SLG). All other cells expressing *dsx^GAL4^* in the CNS appeared to show overlap with the neuronal marker ELAV (Figure 8B,C).

**Figure 9:**
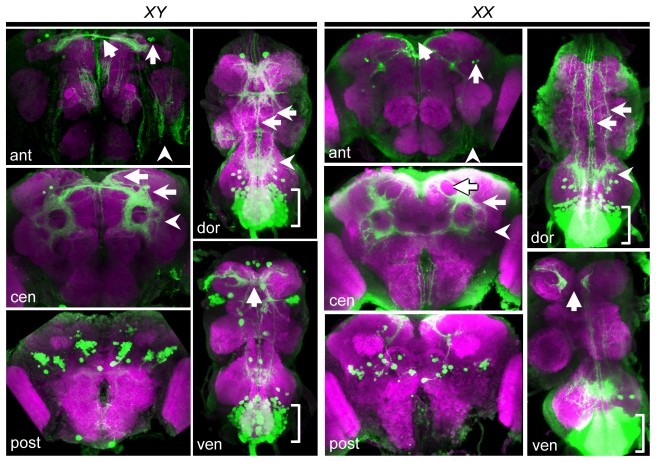
*dsx^GAL4^* is expressed in neurons with sexually dimorphic projections. *dsx^GAL4^* expression in the brains and VNCs of peri-eclosion males (*XY*) and females (*XX*) visualized with *UAS-mCD8::GFP* membrane-bound GFP reporter (green) and counterstained with DN-Cadherin (magenta). Confocal projections of brain and VNC sections are shown: anterior (ant), central (cen), posterior (post), dorsal (dor), and ventral (ven). For each panel, brains are in the left column and VNCs are in the right column. (*XY*) In the male anterior brain, projections from *dsx^GAL4^*-expressing neurons project through the anterior dorsal commissure (arrow), and there is expression in aDN neurons (barbed arrow) as well as putative glia on the anterior cortex in the anteroventral optic cleft (arrowhead). In the central brain, *dsx^GAL4^*-expressing neurons innervate the dorsofrontal cortex (DFC, arrow), the peripeduncular neuropile (PPN, barbed arrow), and the ventrolateral protocerebrum (VLPR, arrowhead). In the posterior brain, clusters of *dsx^GAL4^*-expressing cells are found, as described in Figure 7. In the ventral VNC, projections of foreleg gustatory neurons are visible crossing the VNC midline (arrow), while in the dorsal VNC, projections are seen in midline and lateral tracts (arrows), and there is an arborization in the dorsal metathoracic neuropile (arrowhead). Extensive expression is also seen in the abdominal ganglion (brackets, dor and ven). (*XX*) In the female brain, the *dsx^GAL4^* expression pattern is similar to that of males but with several differences. In the anterior brain, there are no projections through the anterior dorsal commissure. Putative glia were seen as in males (arrowhead). In the central brain, expression in the DFC (black-outlined arrow), PPN (barbed arrow), and VLPR (arrowhead) is reduced relative to males. In the posterior brain, there is a dramatic reduction in the number of neurons relative to males. In the VNC, there is expression in the abdominal ganglion but not the thoracic cell bodies. The dorsal metathoracic arborization is still visible (arrowhead, ven), as are medial and lateral tracts (arrows, dor). Prothoracic gustatory projections are visible (arrow, ven) but do not cross the midline.

In the male VNC, *dsx^GAL4^* expression was observed in the prominent TN1 neuronal cluster, which is derived from the prothoracic ganglion (Jim Truman, personal communication), and in the densely packed neurons of the abdominal ganglion (AbN) (Figure 7). Expression was also seen in single or paired TN2 neurons within each thoracic hemisegment. In contrast, *dsx^GAL4^* was not expressed in the thoracic ganglia of females, while AbN expression was significantly reduced in females through pupal stages before increasing towards eclosion (Figures 7 and 9 and unpublished data). These data are consistent with the reported patterns of DSX immunoreactivity [24],[25].

While previous works reported the position of neuronal cell bodies, the projections of these neurons could not be described by immunolocalization because DSX is a nuclear protein [27]. To determine which areas of the CNS might be influenced by the activity of neurons that express *dsx*, we visualized the projections of *dsx^GAL4^*-expressing neurons throughout the brain and VNC (Figure 9). In the VNC of males, projections from foreleg gustatory neurons were visible in the prothoracic ganglion and showed male-specific crossing of the VNC midline [50]. TN1 neurons were seen to innervate the dorsal pro/mesothoracic neuropile, which may subserve wingsong [26],[55],[56]. The various TN2 neurons also innervated dorsal neuropile regions, while the AbNs innervated the posterior abdominal neuropile and projected through the abdominal trunk nerve (Figure 9).

In the brain, projections from *dsx^GAL4^*-expressing neurons were conspicuously absent from regions of sensory processing or multimodal integration such as the antennal lobes, lateral horn, and mushroom body (Figure 9), many of which are innervated by *fru*-expressing neurons [21],[22],[57]. Projections from *dsx^GAL4^*-expressing neurons were similarly absent from areas subserving memory (e.g., mushroom body, see [58]) and motor patterning (e.g., central complex, see [59]). Instead, *dsx^GAL4^*-expressing neurons projected to and arborized in core neuropile of the central brain, encircling the peduncle and innervating the superior ventrolateral protocerebrum and dorsofrontal protocerebrum (Figure 9) [60]. All four of the posterior *dsx^GAL4^*-expressing clusters contributed to this pattern of innervation in both male and female brains. However, we also observed a male-specific projection through the anterior dorsal commissure, in accord with the morphology reported for *fru*-expressing P1 neurons within the *dsx*-expressing pC1 cluster [51]. In females, no *dsx^GAL4^*-expressing neurons projected across the anterior dorsal commissure, consistent with *dsxF*-mediated cell death of the P1 neurons [51], but a subset of the remaining pC1 neurons contributed to non-commissural projections in the protocerebrum. Taken together, the projection patterns of *dsx^GAL4^* neurons in the brain suggests that *dsx* may regulate sexual dimorphism in neurons that are not simply serving sensory processing or motor output but instead define or modulate a core circuit underlying sexual behavior.

Findings from our work and that of others suggest that *dsx* has several roles within the CNS. First, *dsx* sculpts sexually dimorphic CNS development in several neuronal structures, including the P1 cluster of the posterior brain [51], the TN1 cluster of the mesothoracic ganglion [26] (but see [24]), neurons of the abdominal ganglion [52],[61], and projections of foreleg gustatory neurons in the VNC [50]. In some of these contexts, *dsx* has been shown to sex-specifically regulate neuron number by either prolonging neuronal proliferation [61] or preventing apoptosis [24]. However, we also note that *dsx^GAL4^* is expressed in single neurons that are not obviously sexually dimorphic in number (e.g., pMN1, pMN2, and aDN). Since *dsx^GAL4^* continues to be expressed in both classes of these neurons into adulthood, this implies that *dsx* may play an additional role in regulating sex-specific connectivity or function in these neurons. Lastly, the expression of *dsx^GAL4^* in glia raises the possibility that *dsx* acts to influence brain function through a previously unanticipated mechanism.

## Discussion

Here we report the generation of a targeted insertion of the *GAL4* coding sequence into *doublesex* to generate *dsx^GAL4^*. *dsx^GAL4^* has allowed us to broadly characterize the expression patterns of *dsx* across development in many tissues and provides a tool to investigate the role of *dsx* in particular aspects of sexual development.

Perhaps the most striking feature of our findings is that *dsx* transcription is regulated in a very dynamic and precise temporal and spatial pattern. Temporally, expression of *dsx* begins as early as mid-embryogenesis in the somatic cells of the gonad, whereas in some imaginal tissues it is not expressed until the mid- to late pupal period and persists into adults. Spatially, *dsx* is expressed in nearly all cells of some tissues, but in only a few, or no cells in other tissues. Moreover, even within one imaginal disc there can be very dynamic changes in *dsx* expression. There are several important implications of this diverse and dynamic expression pattern.

First, our findings provide significant insight in to how *dsx* functions through other regulatory genes to orchestrate sexual development. Studies have shown that *dsx* brings about many aspects of sexual development by sex-specifically modulating, in specific cells and tissues, the activities of generic transcription factors and cell–cell signaling molecules that are deployed sex-nonspecifically in other cells and tissues. For example, DSX^F^ negatively regulates the expression of *bnl*, the Drosophila FGF gene, in a subset of cells of the genital disc so that it is not transcribed in females, but is transcribed in males, where DSX^F^ is absent [62]. Male-specific expression of FGF in turn induces genital-disc-associated mesodermal cells that express the fly FGF receptor to migrate into the disc, dedifferentiate into ectoderm, and ultimately produce part of the male internal genitalia [62]. FGF and its receptor also function sex-nonspecifically in other aspects of Drosophila development [54],[63]. Findings such as these raised the question: How is the specificity that allows *dsx* to regulate genes like *bnl* in one tissue, but not another, encoded? Our findings here suggest that some of the specificity of *dsx* function likely comes from the fact that the DSX proteins are deployed in precise patterns across development.

Second, our findings also provide insight into the results of studies with temperature-sensitive sex determination mutants, which revealed that *dsx* likely acts at different times in various cell lineages to determine different aspects of sex [38]. Indeed, even within one cell lineage, different aspects of sex require the functioning of the sex hierarchy at different times [38]. The finding that *dsx* has a highly dynamic temporal expression pattern offers a basis for such observations.

Third, until now, sexual differentiation in flies has been considered to be an adult characteristic. No sexual dimorphisms in purely larval cells have been reported. Thus we were surprised to see that *dsx* is expressed throughout larval fat bodies of very late third instar larvae. If the expression of *dsx* in very late third instar larval fat bodies is reflective of sexual differentiation, we believe this may be related to the fact that larval fat bodies persist until shortly after adult eclosion. Perhaps the remnants of larval fat bodies are utilized during the pupal period or in young adults and the optimal compositions of these materials differ between males and females.

Fourth, basic evolutionary considerations suggest that the highly refined patterns of *dsx* and *fru* transcriptional regulation arose through the piecemeal modification of their cis-regulatory regions. While one focus of evolutionary studies on the Drosophila sex hierarchy has very profitably centered on cis-regulatory regions of genes that are immediately downstream of *dsx* [64],[65], our findings suggest that another rich and complementary evolutionary history with respect to sex in flies resides in the cis-regulatory regions of *dsx* and *fru* that are integral to the elaborate temporal and spatial regulation of these genes.

Finally, and perhaps most importantly, not all cells express *dsx*. This finding has significant implications with respect to the nature of sexuality. Our reasoning is as follows. Most aspects of sexual differentiation in flies are determined cell autonomously, i.e. the functioning of *dsx* and/or *fru* within a cell directs that cell's pattern of sexual differentiation (reviews [1],[66]). Thus, the findings that many cells express neither *dsx* nor *fru* suggest, most simply, that such cells do not differentiate sexually. In that case, Drosophila males and females are mosaics composed of some cells that know their sex (express *dsx* and/or *fru*) and sexually differentiate, and other cells that do not express *dsx* and/or *fru* and thus don't sexually differentiate. A caveat to this simple reasoning comes from more recent findings that in certain cells in Drosophila, sexual differentiation is specified non-autonomously (reviews [2],[67]). In these cases, cells expressing *dsx* direct neighboring cells, via the modulation of cell–cell signaling, to undergo sex-specific differentiation. The neighboring cells that are thus directed may or may not express *dsx*. We look on these cases where local cell–cell interactions specify sexual differentiation as “exceptions that prove the rule.” We believe it is very unlikely that the existence of such local cell–cell interactions offer an alternative explanation to our general conclusion that most cells that don't express *dsx* and/or *fru* don't know their sex (either directly by *dsx*/*fru* expression, or indirectly by a cell–cell interaction of the type just noted). Most simply, many cells that don't express *dsx* are found in large contiguous domains (e.g., all of the embryo except the somatic gonad, large parts of and even entire imaginal discs and their adult derivatives, etc.). Thus we suggest that in flies, both males and females are, in fact, mosaics in which some cells are competent to sexually differentiate and other cells are not.

These findings have also contributed substantially to crystallizing a major revision of our understanding of how sex is specified in Drosophila. In the canonical view of how the sex hierarchy functions, *Sxl* plays a unique, key role as the master regulatory gene, as its activity state (ON in females, OFF in males) is the sole factor dictating whether male or female sexual differentiation occurs in each cell throughout the soma (review [1]). Once the state of *Sxl* activity was determined in response to the embryonic assessment of the X chromosome∶autosome ratio, the autoregulatory function of *Sxl* fixed the splicing milieu in all somatic cells as either male or female throughout development. Downstream of *Sxl* in the sex hierarchy, the *tra* and *dsx* genes were believed to be constitutively expressed, and their expression governed solely by sex-specific alternative splicing. Thus, in this canonical view, there is only one decision point in the hierarchy—setting *Sxl*'s activity state. Further, under this view, the findings that *dsx* controlled many different processes in cell-type specific ways were attributed to there being different arrays of other regulatory molecules with which DSX worked in each of these cell types.

The findings that the expression of the pre-mRNAs encoding *fru*'s sex-specific functions are under precise transcriptional regulation [20], as is the expression of *dsx* in the embryo and CNS (www.fruitfly.org/cgi-bin/ex/insitu.pl) [23]–[27] and indeed in all somatic tissues (this report), have shown that the canonical view of how the fly sex hierarchy functions must be modified. There are, in fact, two levels of regulation governing the expression of sex by a cell. First, the fundamental role of *Sxl* in setting up the competency of all somatic cells to sex-specifically splice the transcripts of the downstream sex regulatory genes is unchanged. However, there is another, previously unrecognized layer of regulation of the hierarchy at the transcriptional level: temporally and spatially regulated transcription of the terminal sex determination regulatory genes, *dsx* and *fru*, results in only some cells having the competency to respond to *Sxl*'s action and produce the sex-specific DSX^F^, DSX^M^, and FRU^M^ transcription factors that confer the potential for sexual differentiation. One particularly satisfying aspect of this revised perspective on the fly sex hierarchy are the striking parallels between the *dsx* and *fru* branches of the hierarchy, which indicate that both branches are likely governed by the same developmental and evolutionary logics.

There is an intriguing caveat to our suggestion that in flies both sexes are mosaics of cells that express *dsx* and/or *fru* and thus have the potential for sexual differentiation, and other cells that don't express *dsx* and/or *fru* and thus cannot undergo sexual differentiation. If the dosage compensation branch of the hierarchy is included in these considerations, then it appears that two different types of sex-regulated functions (universal and tissue-specific) are governed in distinct ways. There are two features of somatic sexual differentiation in Drosophila that probably involve all somatic cells—dosage compensation and body size (females are larger than males). Dosage compensation is controlled directly by *Sxl* via a universal mechanism. How exactly body size is specified is not clear, but it is known that its regulation resides above *tra* in the sex hierarchy [1] and may thus also be controlled via a universal mechanism. In contrast, the tissue-specific aspects of sexual differentiation are jointly specified by *Sxl* splicing regulation and by the transcriptional controls of *dsx* and *fru* expression.

Our revised perspective on sexual development also offers insight into certain aspects of the evolution of sexuality. First, the highly refined temporal and spatial patterns of *dsx* and *fru* expression indicate that these genes have likely evolved by the progressive addition or subtraction of elements from their expression patterns. Second, we know that the genes that respond to *dsx*, either directly or indirectly, seem to be different in each cell type in which *dsx* functions (reviews [2],[64],[65]. Thus evolution has likely acted both (1) to allow the array of genes that are regulated by *dsx* to shift as such genes lose or acquire DSX binding sites and (2) to shift the cells and tissues in which *dsx* functions by altering the cis-regulatory regions of *dsx* that specify the temporal and spatial patterns of its expression. Significantly, evolutionary changes that lead to *dsx* expression in a new cell population open the possibility of *dsx* acquiring novel regulatory targets in those cells. While there is currently much less information on the targets of *fru*, we suggest that the situation is likely the same. Given the fundamental role of *dsx* in fly sexuality, coupled with our finding of the enormous spatial and temporal diversity in *dsx*'s deployment across development, we hope that these findings will stimulate analogous studies of *dsx* homologues in other species.

When we look at how sexual differentiation is controlled in Drosophila today, we are seeing a sum of the evolutionary decisions that led to novel groups of cells or tissues, and particular sets of genes therein, becoming sex-specifically regulated. Strikingly, nearly all the decisions as to how to implement such sexual differentiation were the same—to deploy *dsx* or *fru*. It is reasonable then to wonder why it has consistently been *dsx* or *fru*, rather than some other transcription factor, which was selected to govern a new aspect of sexual development. We think part of the explanation for this observation is that *dsx* and *fru* are already structured for sex-specific alternative splicing, and thus evolution has only to select for either a novel place and/or a new target to come under their regulation. For another transcription factor to fulfill this role, evolution would have to select not only for the same events as just enumerated for *dsx* and *fru* but in addition for the new transcription factor to evolve to be sex-specifically regulated in response to the activity of *Sxl*.

Our current perspective on sexual development in flies is likely to be of significant heuristic value in understanding the processes of sexual differentiation in other species. First, that males and females are both sexual mosaics suggests that there is an evolutionary advantage to only some cells having the competency to sexually differentiate (see also [67]). This may simply indicate that particular cells and tissues acquire *ad hoc* the potential for sexual differentiation (expression of *dsx* and/or *fru*) when there is a selective advantage to the individual. That evolution appears to have taken this route repeatedly in Drosophila suggests that giving all cells the ability to sexually differentiate may negatively impact fitness. In this regard, it is of interest to note that pan-neural expression of FRU^M^ or ubiquitous expression of DSX^M^ is highly detrimental to survival (our unpublished results) [68],[69]. Second, the varied deployment of these factors in otherwise sex-neutral tissues may mediate the diversification of species. Finally, given the extensive commonality across animal species in (1) the genes they possess and (2) how they regulate most fundamental cellular and developmental processes, it seems likely that the mosaic nature in one species of a process as ancient and fundamental as sexual differentiation will likely be representative of sexuality in many other animal species.

## Materials and Methods

### Drosophila Stocks and Culture

Lines *dsx^GAL4^* and *UAS-dsxIR* (*P{WIZ-dsxIR}4 P{WIZ-dsxIR}10*) were generated as described below. w; *P{w[+mC] = UAS-Stinger}2, P{w[+mC] = lexAop-FRT-tdTomato(nls)}6.2*; *fru^P1.LexA^*/*TM6B* is described in [50]. *dsx^1^* and *In(3LR)dsx^M+R13^* are from our lab stocks. Lines from the Bloomington Drosophila Stock Center were: *P{ry^+t7.2^ = 70FLP}11 P{v[+t1.8] = 70I-SceI}2B noc^Sco^/CyO*, *S^2^*, *P{w[+mC] = UAS-mCD8::GFP.L}LL5P*, *{w[+mC] = UAS-RedStinger}4/CyO*, *P{ ry^+t7.2^ = lArB}neur^A101^ ry^506^/TM3*, and *ry^RK^ Sb^1^ Ser^1^*. Lines received as gifts were: *P{w[+mC] = UAS-Stinger}2* (Scott Barolo), *P{lArB}bab1^A128.1F3^* (Frank Laski), and *JFRC-IVSA1* (Barret Pfeiffer). Crosses were performed at 25°C except for inhibitory RNA crosses, which were performed at 29°C.

### Molecular Biology

*dsx* sequences were PCR-amplified using AccuPrime Supermix (Invitrogen) from genomic DNA prepared with the DNeasy Tissue Kit (Qiagen), and sequenced prior to use. 721-bp *dsx* exon 2 fragment was amplified with primers AAGTCACTTACCCAAGGGCACATTG and CCTCCTGAGTCATCACCATCATGTC and used to make p*P{WIZ-dsxIR}* as per [70]. The *dsx* 2.8-kb 5′ and 2.7-kb 3′ homology arms, extending between genomic sequences ATGTACTAGTCCGTCCGTTTGTCTG and CATGATTCCAGCTTCTGATATCCTA, and GAATTCAATTTGCCTCGCTTTAAAT and GGTTTCGGAGGAGAACTGGAATAGC, respectively, were cloned flanking the *GAL4* coding sequence and transferred into p*P*{*WhiteOut2*} (gift of Jeff Sekelsky) to make p*P*{*WO2*-*dsx*-*GAL4*}. Details available upon request.

### Transgensis and Homologous Recombination

p*P*{*UAS*-*dsx*IR} transgenics were made by *P* element-mediated germline transformation using standard methods. Line *UAS-dsxIR4/10* contains two copies of the transgene. p*P*{*WO2*-*dsx*-*GAL4*} transgenics (Rainbow Transgenic Flies, Inc.) were made as described above and four independent integrant lines were isolated to serve as donors of the *dsx^GAL4^* DNA substrate for homologous recombination [29]. Donors were crossed to the line containing heat-shock-inducible FLP recombinase and I-SceI endonuclease transgenes [29] and larvae were heat shocked for 1 h at 37°C on days 3 and 4 of development. ∼5,000 female F1 progeny containing all three elements were crossed to *UAS-mCD8::GFP*, and the F2 progeny screened for candidates with changes in the GFP expression pattern relative to the donors alone. Candidate lines producing intersexual progeny when crossed to *dsx^1^* or *In(3LR)dsx^M+R13^* were PCR-tested using the 5′ genomic and Gal4 primers, GTGTGTGAGGCTGCCTATGTACTAG and ATGCTTGTTCGATAGAAGACAGTAG, and the 3′ genomic and *GAL4* primers CCCATGGTGTCGGTATCTCAAAG and TCACTACAGGGATGTTTAATACCAC, respectively. Using these primer pairs, insert-specific PCR products were generated for the 5′ and 3′ ends of the inserted *GAL4*. White-eyed, *w;dsx^GAL4^* lines were established over the balancer *TM6B*.

### *dsx* Inhibitory RNA

Males from a *w+;dsx^GAL4^* line containing a wild-type *X* chromosome (red-eyed) were crossed to *w;UAS-dsxIR* females to determine the chromosomal sex of intersexual progeny (red-eyed are *XX*, white-eyed are *XY*).

### Tissue Dissection, Staining, and Imaging

Native fluorescence of the UAS-induced reporter proteins was imaged in embryos and imaginal discs, with the exception of the second instar genital disc, which was immunostained with anti-GFP. CNS expression of GFP reporters was also detected with anti-GFP. Larval instars were staged by spiracle morphology [46]. Dissected gonads and imaginal discs were fixed with 4% paraformaldehyde (Electron Microscopy Sciences) in PBS for 15–25 min at 22°C. Blocking and antibody incubations were done in PBS with 0.1% Triton X-100 and 5% normal goat serum (Vector Laboratories) overnight at 4°C or 4–8 h at 22°C. Discs and gonads were mounted in Vectashield mounting media with DAPI (Vector Laboratories). CNS tissues were immunostained largely as described in [71]. Briefly, CNS tissues were fixed for 30 min with 4% paraformaldehyde in PBS, blocked in 4% normal goat serum in TNT, incubated at 4°C overnight with primary or secondary antibodies in TNT alone, and mounted in Fluoromount (Electron Microscopy Sciences). Primary antibodies from the Developmental Studies Hybridoma Bank were used at the indicated dilution: mouse anti-SCR 6H4.1 (1∶20), anti-EN 4D9 (1∶20), anti-Cut 2B10 (1∶20), and anti-EYA 10H6 (1∶25); rat anti-DN-Cadherin Ex#8 (1∶40), anti-Neuroglian BP104 (1∶40), anti-REPO 8D12 (1∶20), and anti-ELAV 7E8A10 (1∶10). Additional antibodies were: rabbit anti-VASA (1∶1000; gift of R. Lehmann) and rabbit anti-GFP (1∶1000 for discs or 1∶800 for CNS; Molecular Probes/Invitrogen); *lacZ* reporter expression was visualized with anti-β-galactosidase (1∶1000, Promega). Alexa Fluor fluorescently conjugated goat secondary antibodies (Molecular Probes/Invitrogen) were used at 1∶500 or 1∶800: 488 anti-mouse, 546 anti-mouse, 568 anti-rabbit, 647 anti-rabbit, and 647 anti-rat. Fluorescence imaging of live embryos and brightfield imaging of adult legs were done on an Axio Imager M1 (Zeiss). All other imaging was done on an LSM510 Meta or LSM710 laser scanning confocal microscope (Zeiss) using 20× air or 40× oil immersion objectives. Confocal slices were manipulated using Image J. Photoshop CS3 software was used to adjust brightness and contrast, as well as to crop images.

## Supporting Information

Figure S1 *dsx^GAL4^* is expressed in genital and thoracic discs. (A) Expression of *UAS-mCD8::GFP* membrane-bound GFP reporter (green) in second instar larval tissue immunostained with anti-GFP. DNA is stained with DAPI (blue). A bilaterally symmetrical group of cells is revealed in the ventral posterior of the larva in the expected location of the genital disc. Note the surrounding larval tissue does not express *dsx^GAL4^*. Scale bar, 10 µm. (B–C) Expression of *UAS-RedStinger* nuclear DsRed reporter (magenta) in genital discs of third instar larva. DNA is stained with DAPI (green). Almost all cells of the male and female discs express *dsx^GAL4^*, with the exception of cells along the lateral edges, as shown for the female disc (arrow). Scale bars, 50 µm. (B) Female. (C) Male. (D) Expression of *UAS-RedStinger* nuclear DsRed reporter (magenta) is compared to the position of cells expressing the proneural marker *neur-lacZ* (green) in the thoracic discs of a mature wandering third instar larva. Shown are discs corresponding to the wing (w), second leg (2l), haltere (h), and third leg (3l). Few cells express *dsx^GAL4^*, although expressing cells are frequently seen near the stalks of the discs (asterisk). There is no overlap with *neur-lacZ*. Scale bar, 500 µm.
(4.20 MB TIF)

Figure S2 *dsx^GAL4^* is expressed in adult adipose tissue and oenocytes. (A) Expression of *UAS-mCD8::GFP* membrane-bound GFP reporter in a sheet of dorsal abdominal adipose cells from an adult male. Single confocal section shown. (B) Expression of *UAS-RedStinger* nuclear DsRed reporter reveals patches of oenocytes around the abdominal spiracles (arrows) of an adult female. Lateral view of live, whole abdomen. Rows of abdominal muscle nuclei are also seen on the ventral abdomen (bracket). Anterior (A), posterior (P), dorsal (D), and ventral (V). Confocal Z projection. (Ci–ii) Expression of *UAS-RedStinger* nuclear DsRed reporter reveals patches of oenocytes under the segmental sternites (brackets) of an adult female. Ventral view of live, whole abdomen. Laterally oriented rows of abdominal muscle nuclei are seen. Large nuclei of a Malpighian tubule are also seen under the abdominal wall (arrows). Confocal Z-projection. (Ci) DsRed alone. (Cii) DsRed (red) merged with cuticle autofluorescence (blue).
(2.38 MB TIF)

Figure S3 *dsx^GAL4^* is expressed in subsets of cells and tissues of adult digestive organs. Cross-sections and superficial views of various digestive organs from a 7-d-old adult female. DNA is stained with DAPI (blue in merge images). Confocal Z projections. (A) Expression of *UAS-RedStinger* nuclear DsRed reporter (*dsx*, red in merge) is seen in a subset of epithelial tissues of the proventriculus (bracket), most prominently in the stomadaeal valve (arrow). Expression is also seen in crop epithelia (barbed arrow). Cross-section shown. (B) Expression of *UAS-mCD8::GFP* membrane-bound GFP reporter (*dsx*, green in merge) in a low magnification, superficial view of the midgut. (C) Expression of *UAS-mCD8::GFP* membrane-bound GFP reporter (green) in a high magnification, superficial view of an anterior portion of the midgut. Large enterocytes (arrow) in this region express *dsx^GAL4^*. (D) Expression of *UAS-RedStinger* nuclear DsRed reporter (red). (Di) Surface of crop epithelium (bracket) with *dsx^GAL4^* expression in a subset of cells. (Dii) Expression is seen in the Malpighian tubules (arrow) but not the hindgut (bracket).
(3.10 MB TIF)

Figure S4 *dsx^GAL4^* is expressed in adult muscles. (A) Expression of *UAS-RedStinger* nuclear DsRed reporter (red) in large muscles of the thorax in a 7-d-old adult female. In the absence of *dsx^GAL4^* (−*dsx^GAL4^*), *UAS-RedStinger* is not expressed. In the presence of (+*dsx^GAL4^*), DsRed (red) is seen in the rows of muscle nuclei. DNA stained with DAPI (blue).
(1.03 MB TIF)

Figure S5 *dsx^GAL4^* is expressed in tissues associated with the genitalia of males and females. Expression of *UAS-mCD8::GFP* membrane-bound GFP reporter (green) and autofluorescence of cuticular elements (magenta) are shown for adult male (A–E) and female (F–J) samples. Confocal Z projections. (A) Seminal vesicle (bracket) and cross-section through ejaculatory duct (ED) (arrow). (B) Ejaculatory bulb (EB). (Bi) Cross-section through EB (bracket) and superficial view of associated muscles (arrow). (Bii) Superficial view of EB (bracket). (C) Terminal epithelium of the testis (arrow) and surface of the ED (bracket). (D) Muscles (arrow) associated with cuticular elements (barbed arrow) of the male genital apparatus. (E) Muscles (arrow) associated with cuticle (apodeme) of the penis apparatus (barbed arrow). Adipose tissue is also seen (asterisk). (F) The common oviduct bifurcates into the lateral oviducts (arrows), which connect to the base of the ovaries (white asterisks). Adipose tissue is also seen (black asterisk). (G) Spermatheca (arrow), a sperm-storing organ, and associated adipose tissue (asterisk). (H) Tracheoles (arrow) associated with the ovary. Surface of ova are magenta. (I) Muscles (m) associated with the paired cuticular vaginal plates (vps). Parallel rows of vaginal teeth bristles (arrows pointing to short bristles that are visible in magenta and black). Epithelium (e) underlying the vaginal plate cuticle. Cross-section of lateroventral view. (J) Muscles (bracket) associated with the cuticular analia dorsal to the vaginal plates. Cross-section.
(5.38 MB TIF)

## Abbreviations

AbN: abdominal ganglion
aDn: anterior-dorsal neuron
APF: after puparium formation
*dsx*: *doublesex*
EYA: *eyes absent* protein
*fru*: *fruitless*
GSO: gustatory sense organ
*neur-lacZ*: *neuralized-lacZ*
pCd: posterior Cells, dorsal
SCR: *Sex combs reduced* protein
SLG: Suboesophageal Lateral Glia
SLN: suboesophageal lateral neuron
SN: suboesophageal neuron
SOG: suboesophageal ganglion
*Sxl*: *Sex lethal*
*tra*: *transformer*
UAS: upstream activating sequence
