# Supplementary figures and images for: Sex and the Single Cell. II. There Is a Time and Place for Sex
Source: PLoS Biol. 2010 May 4;8(5):e1000365. doi: 10.1371/journal.pbio.1000365 (PMC2864297; doi:10.1371/journal.pbio.1000365)

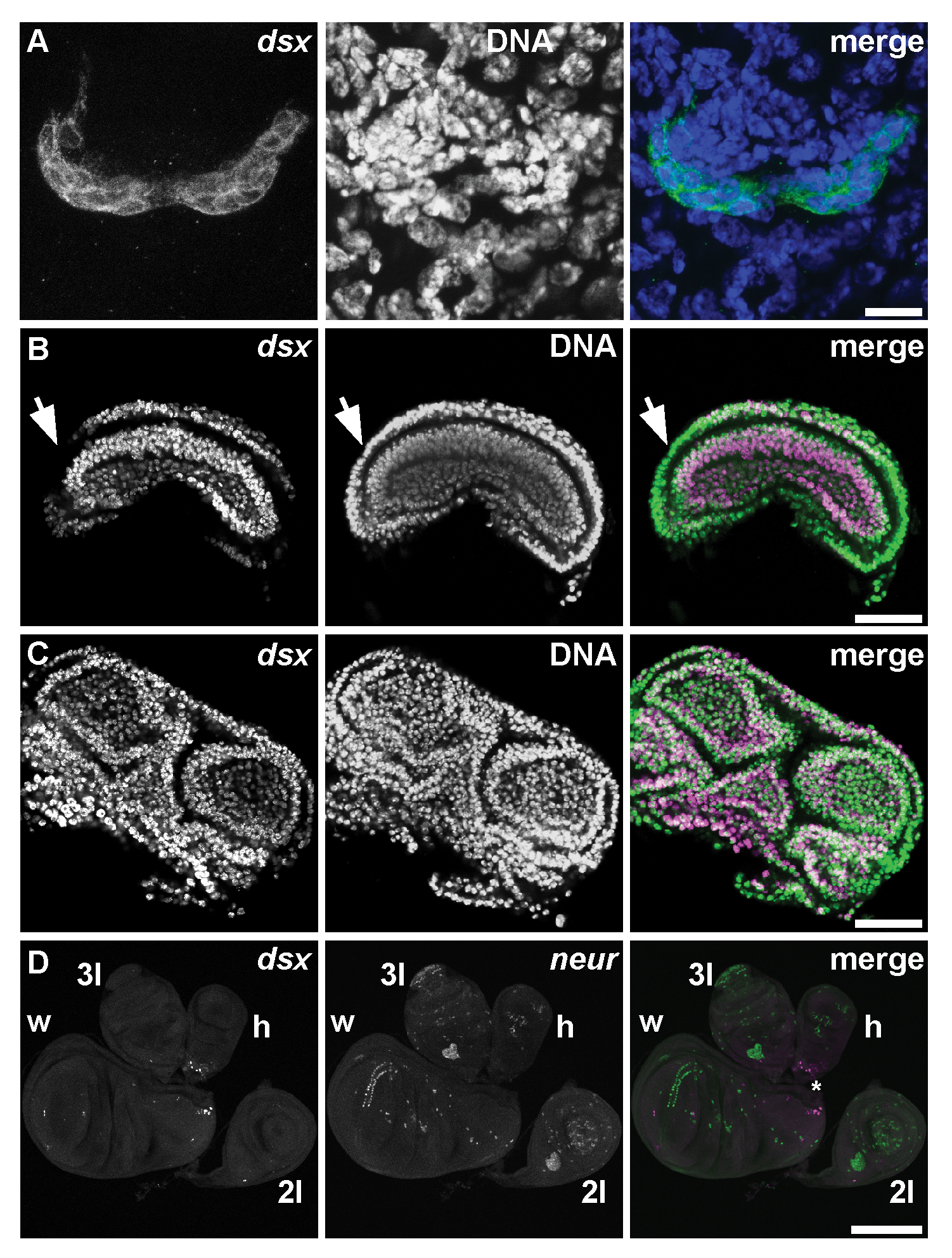

Supplement: Figure S1 — dsxGAL4 is expressed in genital and thoracic discs. (A) Expression of UAS-mCD8::GFP membrane-bound GFP reporter (green) in second instar larval tissue immunostained with anti-GFP. DNA is stained with DAPI (blue). A bilaterally symmetrical group of cells is revealed in the ventral posterior of the larva in the expected location of the genital disc. Note the surrounding larval tissue does not express dsxGAL4. Scale bar, 10 µm. (B–C) Expression of UAS-RedStinger nuclear DsRed reporter (magenta) in genital discs of third instar larva. DNA is stained with DAPI (green). Almost all cells of the male and female discs express dsxGAL4, with the exception of cells along the lateral edges, as shown for the female disc (arrow). Scale bars, 50 µm. (B) Female. (C) Male. (D) Expression of UAS-RedStinger nuclear DsRed reporter (magenta) is compared to the position of cells expressing the proneural marker neur-lacZ (green) in the thoracic discs of a mature wandering third instar larva. Shown are discs corresponding to the wing (w), second leg (2l), haltere (h), and third leg (3l). Few cells express dsxGAL4, although expressing cells are frequently seen near the stalks of the discs (asterisk). There is no overlap with neur-lacZ. Scale bar, 500 µm. (4.20 MB TIF) [file pbio.1000365.s001.tif]

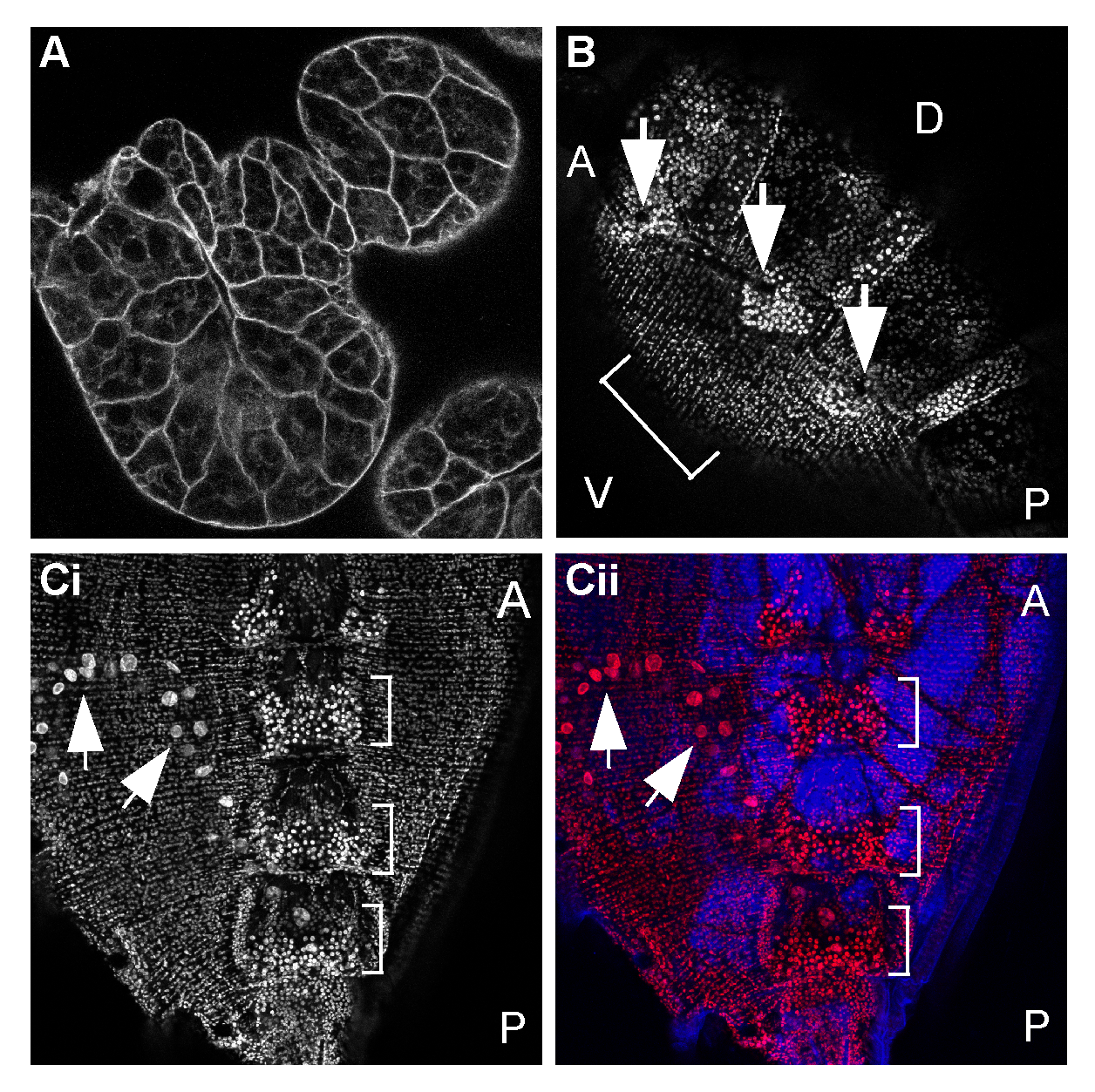

Supplement: Figure S2 — dsxGAL4 is expressed in adult adipose tissue and oenocytes. (A) Expression of UAS-mCD8::GFP membrane-bound GFP reporter in a sheet of dorsal abdominal adipose cells from an adult male. Single confocal section shown. (B) Expression of UAS-RedStinger nuclear DsRed reporter reveals patches of oenocytes around the abdominal spiracles (arrows) of an adult female. Lateral view of live, whole abdomen. Rows of abdominal muscle nuclei are also seen on the ventral abdomen (bracket). Anterior (A), posterior (P), dorsal (D), and ventral (V). Confocal Z projection. (Ci–ii) Expression of UAS-RedStinger nuclear DsRed reporter reveals patches of oenocytes under the segmental sternites (brackets) of an adult female. Ventral view of live, whole abdomen. Laterally oriented rows of abdominal muscle nuclei are seen. Large nuclei of a Malpighian tubule are also seen under the abdominal wall (arrows). Confocal Z-projection. (Ci) DsRed alone. (Cii) DsRed (red) merged with cuticle autofluorescence (blue). (2.38 MB TIF) [file pbio.1000365.s002.tif]

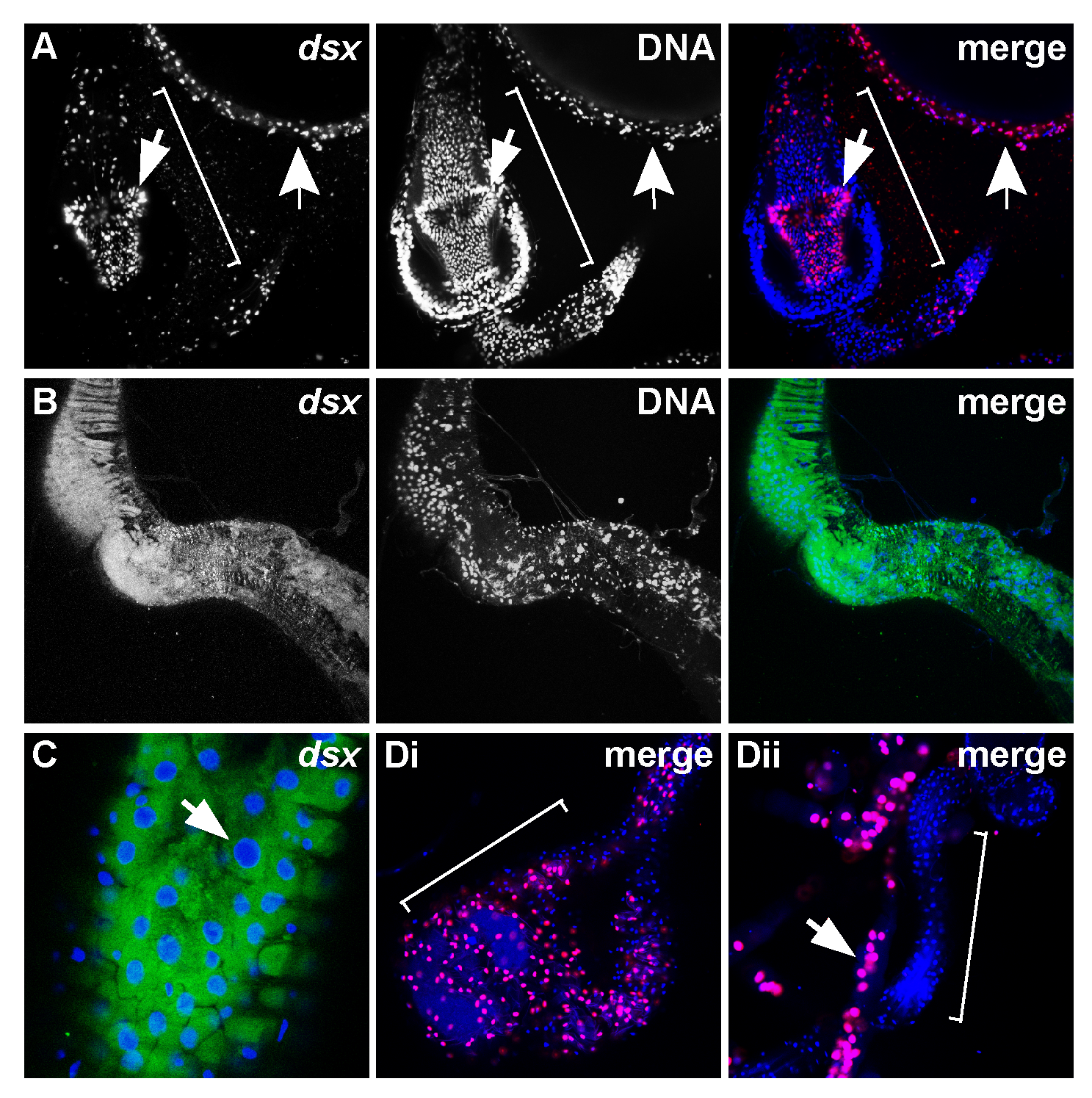

Supplement: Figure S3 — dsxGAL4 is expressed in subsets of cells and tissues of adult digestive organs. Cross-sections and superficial views of various digestive organs from a 7-d-old adult female. DNA is stained with DAPI (blue in merge images). Confocal Z projections. (A) Expression of UAS-RedStinger nuclear DsRed reporter (dsx, red in merge) is seen in a subset of epithelial tissues of the proventriculus (bracket), most prominently in the stomadaeal valve (arrow). Expression is also seen in crop epithelia (barbed arrow). Cross-section shown. (B) Expression of UAS-mCD8::GFP membrane-bound GFP reporter (dsx, green in merge) in a low magnification, superficial view of the midgut. (C) Expression of UAS-mCD8::GFP membrane-bound GFP reporter (green) in a high magnification, superficial view of an anterior portion of the midgut. Large enterocytes (arrow) in this region express dsxGAL4. (D) Expression of UAS-RedStinger nuclear DsRed reporter (red). (Di) Surface of crop epithelium (bracket) with dsxGAL4 expression in a subset of cells. (Dii) Expression is seen in the Malpighian tubules (arrow) but not the hindgut (bracket). (3.10 MB TIF) [file pbio.1000365.s003.tif]

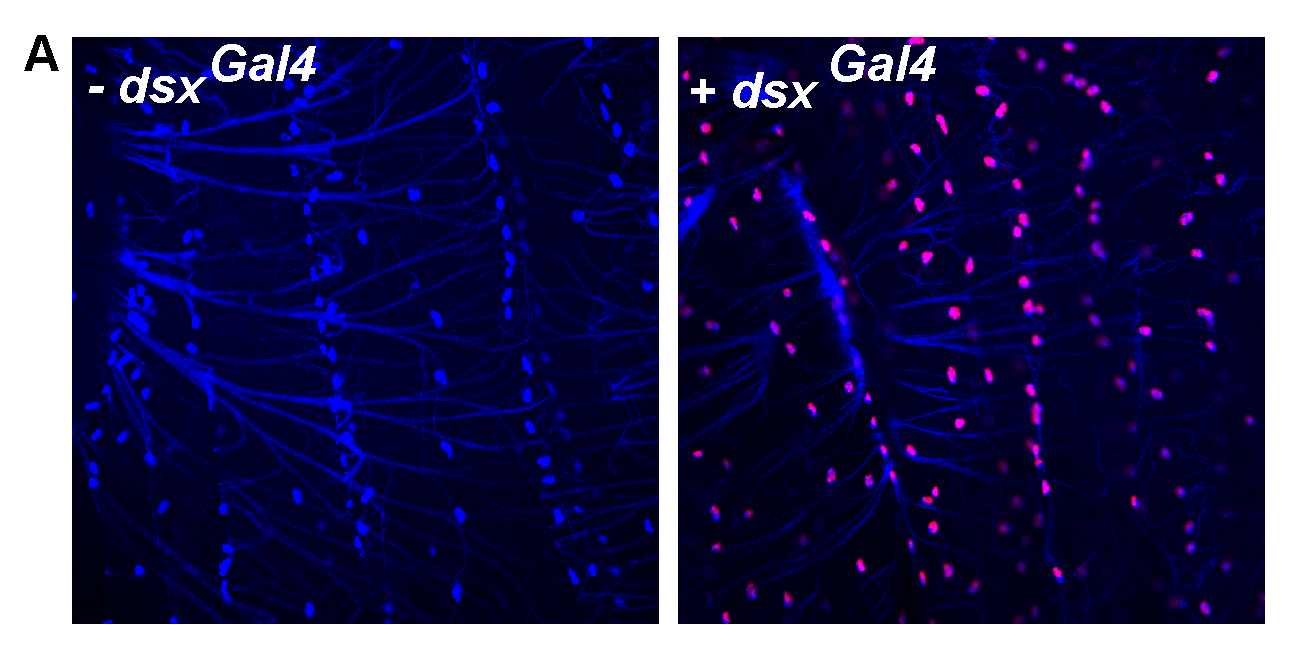

Supplement: Figure S4 — dsxGAL4 is expressed in adult muscles. (A) Expression of UAS-RedStinger nuclear DsRed reporter (red) in large muscles of the thorax in a 7-d-old adult female. In the absence of dsxGAL4 (−dsxGAL4), UAS-RedStinger is not expressed. In the presence of (+dsxGAL4), DsRed (red) is seen in the rows of muscle nuclei. DNA stained with DAPI (blue). (1.03 MB TIF) [file pbio.1000365.s004.tif]

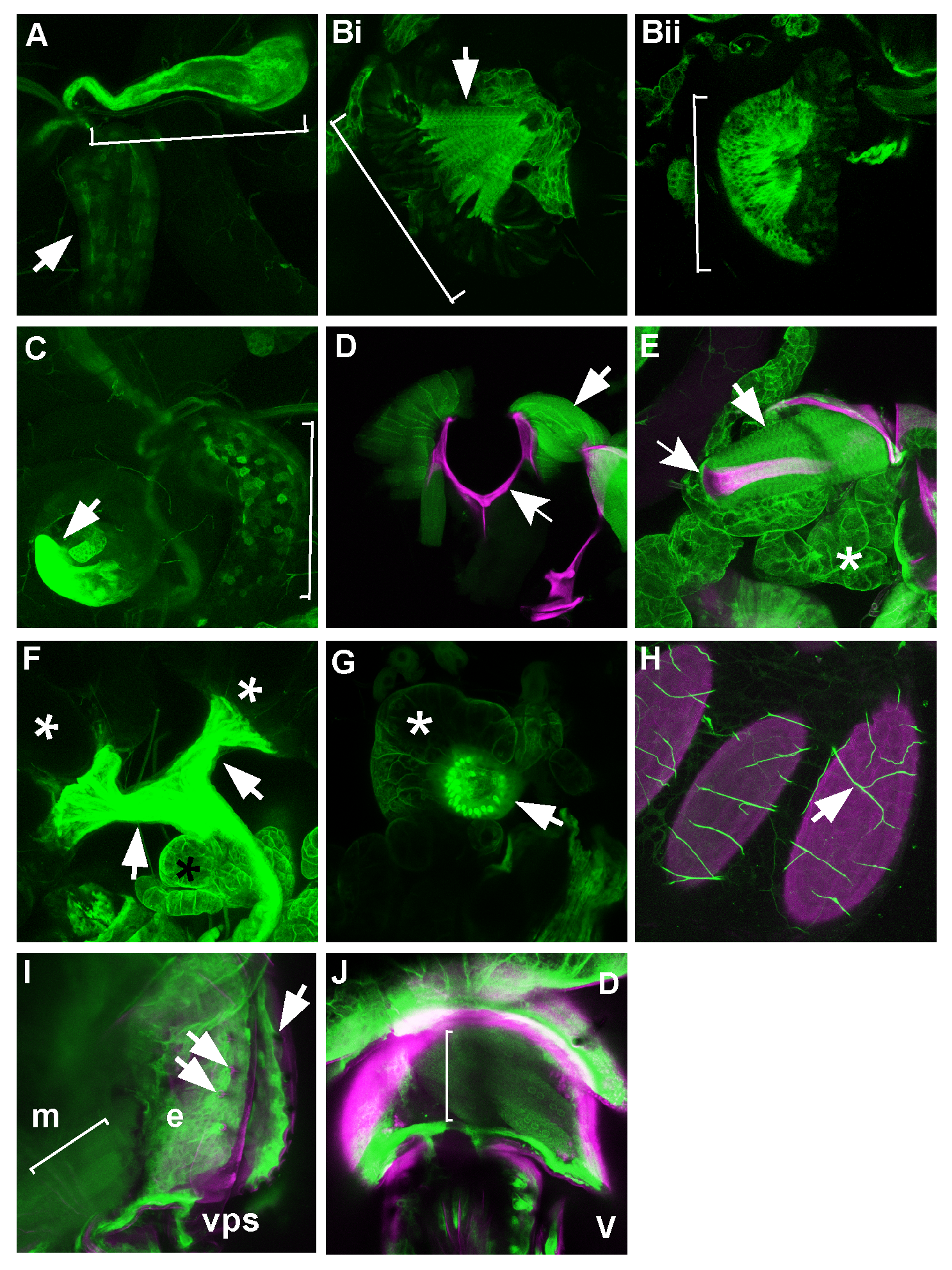

Supplement: Figure S5 — dsxGAL4 is expressed in tissues associated with the genitalia of males and females. Expression of UAS-mCD8::GFP membrane-bound GFP reporter (green) and autofluorescence of cuticular elements (magenta) are shown for adult male (A–E) and female (F–J) samples. Confocal Z projections. (A) Seminal vesicle (bracket) and cross-section through ejaculatory duct (ED) (arrow). (B) Ejaculatory bulb (EB). (Bi) Cross-section through EB (bracket) and superficial view of associated muscles (arrow). (Bii) Superficial view of EB (bracket). (C) Terminal epithelium of the testis (arrow) and surface of the ED (bracket). (D) Muscles (arrow) associated with cuticular elements (barbed arrow) of the male genital apparatus. (E) Muscles (arrow) associated with cuticle (apodeme) of the penis apparatus (barbed arrow). Adipose tissue is also seen (asterisk). (F) The common oviduct bifurcates into the lateral oviducts (arrows), which connect to the base of the ovaries (white asterisks). Adipose tissue is also seen (black asterisk). (G) Spermatheca (arrow), a sperm-storing organ, and associated adipose tissue (asterisk). (H) Tracheoles (arrow) associated with the ovary. Surface of ova are magenta. (I) Muscles (m) associated with the paired cuticular vaginal plates (vps). Parallel rows of vaginal teeth bristles (arrows pointing to short bristles that are visible in magenta and black). Epithelium (e) underlying the vaginal plate cuticle. Cross-section of lateroventral view. (J) Muscles (bracket) associated with the cuticular analia dorsal to the vaginal plates. Cross-section. (5.38 MB TIF) [file pbio.1000365.s005.tif]
